# To Score or Not to Score? A Simulation Study on the Performance of Test Scores, Plausible Values, and SEM, in Regression With Socio-Emotional Skill or Personality Scales as Predictors

**DOI:** 10.3389/fpsyg.2021.679481

**Published:** 2021-10-15

**Authors:** Nivedita Bhaktha, Clemens M. Lechner

**Affiliations:** Department Survey Design and Methodology, GESIS-Leibniz Institute for the Social Sciences, Mannheim, Germany

**Keywords:** socio-emotional skills, non-cognitive skills, large-scale assessments, plausible values, simulation study, scoring, personality assessments

## Abstract

This article addresses a fundamental question in the study of socio-emotional skills, personality traits, and related constructs: “To score or not to score?” When researchers use test scores or scale scores (i.e., fallible point estimates of a skill or trait) as predictors in multiple regression, measurement error in these scores tends to attenuate regression coefficients for the skill and inflate those of the covariates. Unlike for cognitive assessments, it is not fully established how severe this bias can be in socio-emotional skill assessments, that is, how well test scores recover the true regression coefficients — compared with methods designed to account for measurement error: structural equation modeling (SEM) and plausible values (PV). The different types of scores considered in this study are standardized mean scores (SMS), regression factor scores (RFS), empirical Bayes modal (EBM) score, weighted maximum likelihood estimates (WLE), and expected a posteriori (EAP) estimates. We present a simulation study in which we compared these approaches under conditions typical of socio-emotional skill and personality assessments. We examined the performance of five types of test scores, PV, and SEM with regard to two outcomes: (1) percent bias in regression coefficient of the skill in predicting an outcome; and (2) percent bias in the regression coefficient of a covariate. We varied the number of items, factor loadings/item discriminations, sample size, and relative strength of the relationship of the skill with the outcome. Results revealed that whereas different types of test scores were highly correlated with each other, the ensuing bias in regression coefficients varied considerably. The magnitude of bias was highest for WLE with short scales of low reliability. Bias when using SMS or WLE test scores was sometimes large enough to lead to erroneous research conclusions with potentially adverse implications for policy and practice (up to 55% for the regression coefficient of the skill and 20% for that of the covariate). EAP, EBM, and RFS performed better, producing only small bias in some conditions. Additional analyses showed that the performance of test scores also depended on whether standardized or unstandardized scores were used. Only PV and SEM performed well in all scenarios and emerged as the clearly superior options. We recommend that researchers use SEM, and preferably PV, in studies on the (incremental) predictive power of socio-emotional skills.

## 1. Introduction

Assessing socio-emotional skills (also known as “non-cognitive skills,” “twenty-first century skills.” “character strengths,” or “soft skills”)^1^ is becoming increasingly common in large-scale assessment surveys (LSAS) and beyond (Abrahams et al., 2019; Lechner et al., 2019). For example, the OECD has recently devoted an entire study on this issue—the Study on Social and Emotional Skills (SSES; e.g., Kankaraš and Suarez-Alvarez, 2019). Most LSAS now contain selected socio-emotional skills, personality traits, and related constructs in addition to *cognitive* skills, which traditionally have been the focus of LSAS. This surge in research interest is accompanied by a growing interest in socio-emotional skills from policymakers and practitioners and is further fueled by findings suggesting that socio-emotional skills are increasingly in demand in the labor market (Deming, 2017; Allen et al., 2020).

Pertinent studies often examine socio-emotional skills as *predictors* of outcomes such as school achievement, career success, participation in further education, or health (e.g., Roberts et al., 2007; Lechner et al., 2017; Rammstedt et al., 2017; Laible et al., 2020). Moreover, akin to many other research areas (Aiken and West, 1991; Westfall and Yarkoni, 2016; Sengewald et al., 2018), it is routinely of importance to examine whether socio-emotional skills incrementally predict an outcome above and beyond covariates such as cognitive skills, socioeconomic status, or other established predictors of that outcome (e.g., Roberts et al., 2007; Rammstedt et al., 2017; Bergner, 2020; Harzer, 2020; Wagner et al., 2020). That is, such studies are intent on demonstrating the (incremental) predictive validity of socio-emotional skills for consequential life outcomes, which is then taken as evidence for the relevance of socio-emotional skills.

A problem shared by studies on the (incremental) predictive validity of socio-emotional skills is that the skills and traits in question are unobserved (latent) variables that can only be measured indirectly through a set of observed indicators^2^. As a result, the true skill of each individual test taker is, by definition, unknown. Any individual point estimates of that skill—conventionally known as “test scores” or “scale scores”—are but estimates and invariably contain measurement error (see Lechner et al., 2021, for an overview). The most common (though not the only possible) consequence of measurement error is that the regression coefficient of that skill will be attenuated (i.e., biased downward; Fuller, 2006)^3^. Conversely, regression coefficients for covariates are typically overestimated (i.e., biased upward) if measurement error in the skill is unaccounted for (see Westfall and Yarkoni, 2016). Measurement error in the skill estimates can also bias the regression coefficient of the covariates such that the attenuation bias increases as the reliability of the skill decreases (Aiken and West, 1991; Sengewald et al., 2018). The biases in regression coefficients from using fallible test scores can be large enough to lead researchers to erroneous conclusions regarding the predictive power of the skill or its incremental predictive power over a covariate.

Although these problems are generally recognized (at least among methodologists), it is not fully clear just how serious and consequential such bias in regression coefficients from using fallilble test scores may be in studies on the predictive power of socio-emotional skills. In turn, it is not fully clear whether using one of the two theoretically superior options that account for measurement error and eliminate attenuation bias—structural equation modeling (SEM) and plausible values (PV)—are worth the extra effort. Relatively little is known about the performance of different types of test scores, SEM, and PV specifically in relation to socio-emotional skill or personality assessments. This is because most psychometric research has taken place in the context of cognitive assessments that differ in several important regards from socio-emotional skill assessments.

In this study, we present a comprehensive simulation study in which we compare the performance of five different types of test scores, SEM, and PV in scenarios where the focus is on the predictive power of socio-emotional skills in a regression. We designed our simulation study to closely mimic the properties of real socio-emotional skill assessments. In the following, we briefly explain the three main approaches to analyzing skill measures and review prior simulations comparing their performance. We then present our own simulation study and draw on its results to derive recommendations for researchers involved in the study of socio-emotional skills.

## 2. Three Approaches to Analyzing Data From Skill Assessments

There are three principal options for analyzing data from multi-item scales^4^ designed to measure socio-emotional skills and related constructs: Computing test scores (or using pre-computed test scores) and incorporating these test scores in analyses—in the same way as any other observed variable is incorporated; using SEM to model the relationship among the skill and its outcomes or predictors; and incorporating the skill in the form of plausible values (PV) in the regression. As shown in Table 1, these three options differ fundamentally with regard to accounting for measurement error in the skill (*fallibility*); their ease of use (*usability*); and the extent to which analysis results can change depending on factors such as the variables included in the analysis, the subsample used, or the estimator chosen (*immutability*). Next, we briefly review these approaches. For a more in-depth treatment, we refer the reader to Lechner et al. (2021).

**Table 1:** Evaluation of three main approaches to analyzing skill data.

**Method**	**Variants**	**Fallibility**	**Usability**	**Immutability**
Test Scores	▪ Sum scores (weighted, unweighted)	▪ ME not (fully) controlled (−)	▪ Sum scores: Very easy to compute (+)	▪ Sum scores: Immutable across sub-samples, analyses, and analysts (+)
	▪ CTT factor scores (Bartlett, Regression)	▪ Biased standard errors of the latent variable in regressions (−)	▪ Computation requires knowledge of psychometric models but is fairly easy (+)	▪ Factor scores/ability estimates: Immutable if estimates are included with LSAS data (+)
	▪ IRT ability estimates (WLE, MLE, EAP, and MAP)	▪ Biased variance estimates (e.g., underestimation for EAP, overestimation for WLE) (−)	▪ Very easy to use in analysis (+)	▪ Factor scores/ability estimates: Not immutable if estimates are user generated (−)
		▪ Factor score indeterminacy (−)		
Structural Equation Modeling (SEM)	▪ Regular SEM	▪ ME controlled (+)	▪ Requires specialized statistical software (−)	▪ Immutable with fixed measurement model parameters (+)
	▪ IRT-SEM	▪ Unbiased estimates of correlations, means etc. of the latent variable (+)	▪ Requires additional psychometric expertise (−)	▪ Not immutable with free measurement model parameters across sub-samples, analyses, and analysts (−)
	▪ MESE	▪ Measurement model sensitive to model (mis-) specification (−)		
Plausible Values (PV)		▪ ME controlled (+)	▪ User-generated PV require statistical and programming and expertise (−)	▪ Immutable if PV are included with LSAS data (+)
		▪ Approximately unbiased estimates of correlations, means etc. of the latent variable (+)	▪ Using PV in secondary analysis requires basic knowledge of multiple imputation methodology (−)	▪ Not immutable if PV are user generated (−)

*Note: ME, measurement error*.

### 2.1. Test Scores

Test scores (or, equivalently, scale scores) are familiar to researchers working with multi-item tests or scales. There are many different types of test scores that range from simple sum or mean scores—by far the most frequently used type of score—to more complex Bayesian scoring techniques. Test scores are what would be reported back to individual test-takers in assessments that serve practical purposes (e.g., selection or placement). By contrast, in research, the interest is usually not in individual test-takers but in population quantities such as the mean and variance of the skill or the skill's relation to an outcome (Braun and von Davier, 2017). In this regard, all types of test scores share one important limitation that is often overlooked and that renders them a sub-optimal choice for research into skills: Test scores are only *estimates* of an individual's true score; as such, they are fallible (i.e., contain measurement error). This applies to both simple and more complex scoring techniques.

The error variance that tarnishes test scores is likely to lead to attenuation bias when using them as predictors in multiple regression (Fuller, 2006; Schofield, 2015; Braun and von Davier, 2017; Lechner et al., 2021)—a scenario that is ubiquitous in current studies (e.g., Roberts et al., 2007; Bergner, 2020; Harzer, 2020; Wagner et al., 2020). Moreover, it may lead to false positive or false negative conclusions about incremental validity (e.g., Westfall and Yarkoni, 2016; Sengewald et al., 2018). When measurement error in a variable is unaccounted for, the regression coefficients for another variable can be inflated compared with their true population values. Depending on whether the variable is the focal predictor (i.e., a variable whose incremental validity over another is in question) or the covariate (i.e., a variable against which the incremental validity of the focal predictor is being tested), this can lead to false positive or false negative conclusions about incremental validity. Simulation studies have demonstrated that even small amounts of measurement error in the predictor variables can have deleterious effect on parameter estimates, leading to incorrect incremental validity claims (e.g., Westfall and Yarkoni, 2016; Sengewald et al., 2018). Despite this important drawback, test scores are the most widely used method of analyzing data from multi-item tests or scales (for additional drawbacks, see von Davier, 2010; Beauducel and Leue, 2013; McNeish and Wolf, 2020).

### 2.2. Structural Equation Modeling (SEM)

**Figure 1:**
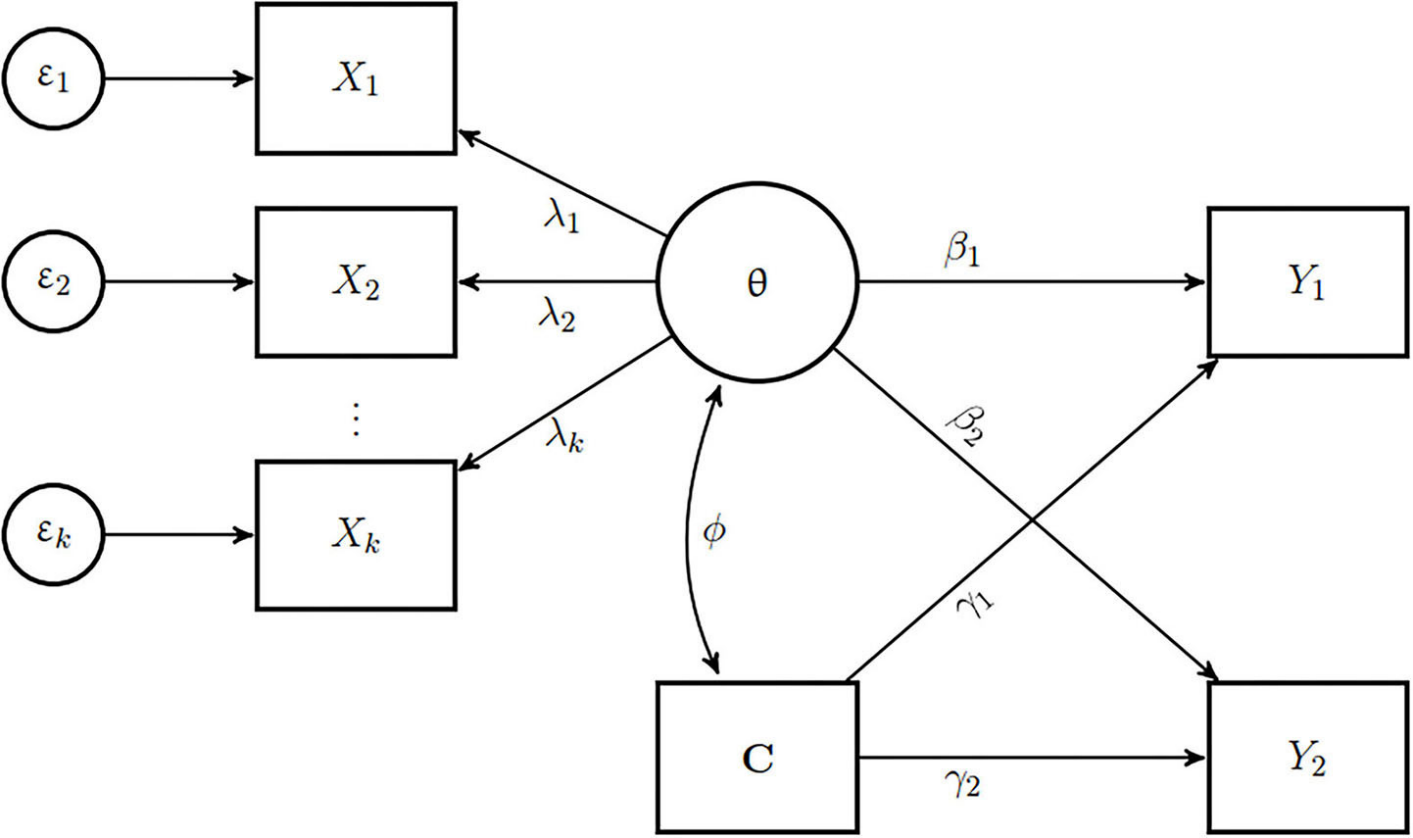
Example of a model used for generating data for the simulation study. The latent skill θ is measured by *k* manifest items *X*_1_, …, *X*_*k*_. The different factor loadings λ_1_, …, λ_*k*_ and measurement error terms ε_1_, …, ε_*k*_ reflect different degrees to which each item reflects the latent ability. *C* is a covariate that has a correlation of ϕ with the skill. Both θ and *C* are predictors of two outcomes *Y*_1_ and *Y*_2_. β_1_ and β_2_ are regression coefficients of θ and γ_1_ and γ_2_ are the regression coefficients of *C* for *Y*_1_ and *Y*_2_ respectively.

SEM is the traditional solution for the problem of measurement error. Instead of computing fallible point estimates of ability from a measurement model, SEM combines a measurement model—typically a classical test theory (CTT) model such as the tau-congeneric model—with a structural model. The measurement model represents the skill as a latent variable that is free from measurement error, and the structural model relates this error-free latent variable to predictors, outcomes, or covariates through regression or correlation paths. This is diagrammatically represented in Figure 1. Notably, respondents' test scores do not appear anywhere in SEM, which can in theory be estimated based on a variance—covariance matrix alone. Hybrid approaches that combine an item response theory (IRT) type measurement model with SEM and mixed effects structural equations (MESE) models have been proposed (Lu et al., 2005; Junker and Schofield, 2012) to allow conditioning the latent variable on covariates in the structural model to reflect extraneous influences on the latent skill. Moreover, item factor analysis (IFA) models, a hybrid approach that uses weighted least squares (WLS) estimator are gaining in popularity (Wirth and Edwards, 2007).

The use of SEM for socio-emotional skills and similar constructs has been propagated in educational and psychological research, especially for the purpose of testing (incremental) predictive power (Westfall and Yarkoni, 2016; Sengewald et al., 2018). Even so, SEM is far from universally used, and researchers outside these fields are typically unfamiliar with this methodology. Moreover, as noted in Table 1, accurate implementation of SEM requires specialized software and psychometric expertise which further limits its usability.

### 2.3. Plausible Values (PV)

Originally developed in the context of cognitive assessments (Mislevy, 1991), PV methodology takes a fundamentally different approach. The basic idea is to treat the latent skill variable as what it inherently is: a missing data problem. Instead of estimating a single test score per respondent, multiple imputations of respondents' unobserved true ability are generated based on a measurement model, the response pattern, and often a set of additional variables that predict the latent skill θ. The possibility to incorporate information from a range of “background” or “conditioning” variables in estimating the PV makes this methodology particularly well-suited for LSAS that use incomplete block designs (also called “planned missingness designs”) in which respondents answer only a subset of the test items for the skill. A set of PV (typically, 5 or 10 per respondent) are then generated by drawing from the posterior distribution of the skills. These PV must not be confused with test scores or the latent variable-they are “best guesses” about the individual's true skill based on a model and are not the skill itself. The variation across the different PV adequately reflects the uncertainty about the respondent's true ability (Braun and von Davier, 2017). The resulting PV are incorporated in the analysis using standard multiple imputation methodology (see Little and Rubin, 2002; Enders, 2010). In this way, PV methodology provides unbiased estimates of population means and variance of the skill, as well as of regression coefficient when the skill is a predictor (Wu, 2005; Braun and von Davier, 2017)^5^. For introductions to PV methodology, see Wu (2005), von Davier et al. (2009), Braun and von Davier (2017), and Lechner et al. (2021). We provide further computational details about PV in the section 5.

PV are ideally suited for LSAS, where the interest is in population quantities (e.g., mean-level differences of the skill across gender), not in individual test-takers. PV are increasingly becoming standard in cognitive LSAS (Braun and von Davier, 2017; Laukaityte and Wiberg, 2018). However, they are seldom used for analyzing personality or socio-emotional skills data, likely because researchers are unaware of the problems of test/scale scores and because both generating and working with PV requires expertise in IRT and missing data analysis.

## 3. What Do We Know About the Performance of the Three Approaches in Studies on Socio-Emotional Skills?

Methodological research on the three methods for analyzing skill scales has almost entirely been motivated by, and taken place in the context of, *cognitive skill* assessments such as TIMMS, PISA, NAEP (Mislevy et al., 1992). However, socio-emotional skill assessments differ in three main ways from traditional cognitive assessments (outlined below). Previous simulation studies have rarely investigated scenarios that are typical of socio-emotional skill assessments (refer Table 2). Moreover, they have mostly focused on only one or two specific approaches (e.g., PV vs. WLE) but have not provided comprehensive comparison of the different approaches to analyzing skill data. As a consequence, it is unclear whether common guidelines and best practices for analyzing skill measures and incorporating them as predictors in regression that were originally derived for cognitive assessments equally apply to socio-emotional skills, personality, and related constructed.

**Table 2:** A brief summary of simulation study articles comparing different approaches to analyzing skills data in the context of cognitive assessments.

**Article**	**Methods compared**	**Number of items**	**Conditions**	**Sample size**	**Replications**
Wu (2005)	WLE, MLE, EAP, PV (1, 2, 3, 4, 5)	3, 20	Varying item difficulty, item descrimination, and ability	2,000	100
Lu et al. (2005)	IRT-SEM, EAP, Standardized NR Scores	10, 20, 30	Varying coefficients of determination in measurement models	300, 500, 800, 2,000	1,000
Monseur and Adams (2009)	MLE, Corrected MLE, WLE, EAP, EAP with conditioning, PV (1, 5), single estimate of PV	3, 5, 20, 50, 100	Varying item difficulty and item descrimination	2,000	Chosen so SE = 0.005
von Davier et al. (2009)	PV (5), Average of all PV, EAP, WLE	8, 16, 24	2 background variables	4,000	Not reported
Estabrook and Neale (2013)	approx. factor score, Bartlett score, Full ML, Unweighted ML	3, 5, 10, 20	Varying factor loadings, missing data conditions	100, 200, 500	10
Aßmann et al. (2014)	EAP and PV	10	Varying item difficulty, item descrimination, and ability. 3 background variables	2,000	200
Borgatto et al. (2015)	WLE, EAP, MAP	15, 30, 45, 60	Varying item difficulty, item descrimination, and ability	1,000	Not reported
Laukaityte and Wiberg (2017)	PV (1, 5, 7, 10, 20, 40, 100), WLE, MLE, EAP	20, 40	Varying mean proficieny and item parameters	4,000, 8,000	30 and 100
Bibby (2020)	PV (3, 5, 10, 15, 20)	10, 20, 40, 60, 80	Varying regression coefficients, population ditribution, and Latent means. Inclusion and exclusion of background variables.	200; 2,000; 10,000	1,000

### 3.1. Socio-Emotional Skill Assessments Differ From Cognitive Skill Assessments

#### 3.1.1. Response Format

With few exceptions, socio-emotional skill and personality items use rating scales in which there are no “correct” responses but different degrees of agreement, intensity, or frequency. To illustrate, the Big Five Inventory-2 (BFI-2; Soto and John, 2017) uses a fully labeled five-point scale (1 = *disagree strongly*; 5 = *agree strongly*). Cognitive assessments, by contrast, often use dichotomous test items (correct/incorrect) or multiple choice items that are then often dichotomized.

Different response formats, of course, entail different levels of measurement (e.g., dichotomous vs. ordered-categorical or “polytomous”) and distributions of the response variables (e.g., binomial vs. multinomial or normal). They also require different modeling approaches (e.g., Rasch models for dichotomous items vs. confirmatory factor analysis or graded response models for rating scales).

#### 3.1.2. Number of Items

Socio-emotional skill and personality scales almost invariably comprise of fewer items than cognitive skill scales. As researchers working with such scales can attest, it is challenging to create unidimensional scales with more than 6 or 8 items. Although additional items increase scale reliability, adding items can also introduce additional sources of (co-)variance that compromise unidimensionality. For example, statements such as “I am good at controlling my emotions” contain more than one source of (co-)variation, such that adding more items often introduces (residual) covariances or secondary factors (merely because some items use the same keyword or grammatical construction). Moreover, many short scales achieve reliabilities and predictive validities that rival those of longer scales (Thalmayer et al., 2011; Rammstedt et al., 2021), tempering the need for (theoretically advantageous) longer scales.

Therefore, socio-emotional skill scales typically use between 4 and 8 items per skill or facet. Longer scales are rare. For example, the BFI-2 (Soto and John, 2017) comprises 15 facets, each measured with 4 items. When aggregated to the Big Five, each dimension comprises 12 items (Soto and John, 2017). OECD's recent SSES (Kankaraš and Suarez-Alvarez, 2019) uses 8 items per facet/skill, the Values in Action Inventory (VIA; du Plessis and de Bruin, 2015) has 7–14 items per skill, and the new behavioral, social, and emotional skills inventory (BESSI; Soto et al., 2021) has 6 items for each of 32 skills.

By contrast, cognitive assessments tend to have more than 20 items per unidimensional contructs (TIMMS, PIRLS, PIAAC, NAEP). As Table 2 shows, most (but not all) of the previous simulation studies on scoring approaches have focused on larger number of items that are typical of cognitive assessments. These studies have also shown that the performance of some of the scoring methods typically improves as the number of items increases. It is not fully clear how the approaches perform when applied to the short scales typical for socio-emotional or personality assessments.

#### 3.1.3. Relation Between Indicators and Latent Constructs

Socio-emotional skill scales rarely follow a tau-equivalent or 1-PL IRT measurement model in which all items have identical factor loadings (in CTT logic) or item discriminations (in IRT logic), respectively. Instead, the size of the factor loadings or item discriminations typically differs between items on the same scale. Generally, for such scales, the items on a unidimensional scale tend to have mixed factor loadings with most items having moderate, few items having high, and some items having low factor loadings. Higher factor loadings can be expected if the scales are widely used and well validated, and if their content is more homogeneous. Loadings can also vary when the scale is applied in different subpopulations that interpret some of the items differently. In contrast, items in cognitive assessments developed using IRT tend to have higher and more similar item discriminations.

The size and homogeneity of loadings/discriminations is an important consideration for scoring because it determines the scale's reliability (in CTT) and the standard error of the test score (in IRT). Put simply, lower reliability implies a higher amount of measurement error in test scores, which in turn determines (typically impairs) how well the test score performs as a predictor in regression. Some of the previous simulation studies on the topic have varied item discrimination or factor loadings to examine how doing so affects the relative performance of scoring approaches (Table 2).

### 3.2. Previous Simulation Studies Rarely Compared All Three Approaches

Many of the guidelines or recommendations for analyzing cognitive assessments were informed from simulation studies comparing contemporary methods with newer methods such as PV. Table 2 presents a brief description of simulation studies that have compared different approaches of utilizing items from cognitive assessments. We can see that there is rich literature on comparing IRT based methods of scoring with PV. However, none of the studies have compared across both IRT and CTT based scoring methodologies and contrasted them with the most widely used method of scoring — mean scores. Some of the earlier simulation studies have considered smaller number of items per scales, yet most of the studies have focused on larger number of items per scale that far exceed the typical number of items for socio-emotional skill scales. As expected, most of the studies have varied the item difficulty, item discrimination, and ability levels. Moreover, most of the studies have compared the different methods for large sample sizes.

Table 3 provides a brief summary of the results of the previous simulation studies. When PV were compared with other methods, PV performed the best in terms of lower bias in variance estimation and standard error. Some of the studies mentioned that EAP and other methods performed well and their performance were comparable to each other in certain cases. Most of the studies indicated that the bias of the test scores reduced with increase in test length (number of items). Sample size seemed to have little bearing on the results. Some of the studies found that the performance of WLE improved drastically with increase in number of items.

**Table 3:** Results of simulation studies conducted by articles considered in Table 2.

**Article**	**Methods compared**	**Results**
Wu (2005)	WLE, MLE, EAP, PV (1, 2, 3, 4, 5)	PV performed better than WLE, MLE and EAP estimates, in recovering population parameters such as the mean, variance, and percentiles, even with very short tests. The bias in WLE and MLE variance estimates increased as test length decreased.
Lu et al. (2005)	IRT-SEM, EAP, Standardized NR Scores	IRT-SEM generated consistent regression parameter estimates for larger sample sizes. EAP and standardized NR scores required >30 test items to attain acceptable finite item bias. Performance of NR and EAP scores were highly comparable regardless of test length and measurement model precision.
Monseur and Adams (2009)	MLE, Corrected MLE, WLE, EAP, EAP with conditioning, PV (1, 5), single estimate of PV	PV was most appropriate while MLE and WLE provided poor variance estimates. EAP with conditioning provided better estimates of variance. Bias in WLE reduced for more than 20 items. Single estimates of PV performed similar to EAP.
von Davier et al. (2009)	PV (5), Average of all PV, EAP, WLE	All methods were similarly close to true value for means. For standard deviation, PV with correct usage was the only consistent method, especially as the number of items on the test decreased. WLE was biased toward more extreme values, while EAP was biased toward the mean.
Estabrook and Neale (2013)	approx. factor score, Bartlett score, Full ML, Unweighted ML	The four scores had negligible differences in case of complete data. Full ML method outperformed other methods in case of missing data.
Aßmann et al. (2014)	EAP and PV	EAP and PV performed well with the MCMC approach with respect to the error and coverage rate, for partially observed background variables even with a relatively large amount of missing values.
Borgatto et al. (2015)	WLE, EAP, MAP	EAP with a uniform prior distribution and WLE method had best performance. WLE performed well especially in scale region where test provided little information.
Laukaityte and Wiberg (2017)	PV (1, 5, 7, 10, 20, 40, 100), WLE, MLE, EAP	PV-based estimates had better recovery of population parameteres than any point estimators. More stable and reliable estimates were obtained at 10 or more PV. Diffferences among the methods were quite small.
Bibby (2020)	PV (3, 5, 10, 15, 20)	Bias in parameters estimates and SE reduced with longer test length and increased sample size. No significant effect on the bias in parameter estimates were observed due to the increase in number of PV.

Hence, despite the important insights offered by previous simulation studies, it is evident from Table 2 that there are some gaps in the current literature on analyzing cognitive assessments, and extant findings cannot be safely generalized to personality or socio-emotional skills assessments. There is a dearth of simulation studies comparing popularly used mean scores, other CTT and IRT based test scores to SEM and PV for scenarios that are typical for socio-emotional skill and personality assessments: small number of items especially with greater variability in factor loadings (or item discrimination) in both small and large sample settings. There are hardly any simulation studies that discuss the performance of different types of test scores in the context of regression analyses in which the skills are used as predictors. Although it may well be the case that the recommendations derived for cognitive skill assessments hold true for socio-emotional skill assessments as well, the distinctions in the nature of the scales and items, and the gaps in the literature, highlights the need for a comprehensive and rigorous examination of the performance of the different approaches specifically in the context of socio-emotional skill assessments.

## 4. Aims and Research Questions of the Present Study

In this study, we present a comprehensive simulation study in which we assess the performance of the three principal approaches to analyzing skills as predictors in multiple regression (different types of test scores, SEM, and PV) under conditions that are typical for socio-emotional skill and personality assessments. We compare the performance of these approaches with regard to two outcomes. The first is the bias in the regression coefficient of the skill when the skill is used to predict an outcome. The second is the bias in the regression coefficient of a covariate in the same model, which relates to questions about the incremental validity of the skill over the covariate (or vice versa). We chose the conditions in our simulation (e.g., number of items, factor loadings, relative strength of the relationship of the skill with the outcome, sample size) to mimic realistic analysis scenarios for socio-emotional skill assessments as closely as possible (for details, see section 5). We address the following research questions:

- How well do the three different approaches (i.e., different types of test scores, SEM, PV) recover the true population values of the regression coefficients of the skill and a covariate? In particular, how large is the bias that may ensue from using fallible test scores?
- How do differences in factor loadings (or item discriminations), the number of items, relative strength of the relationship of the skill with the outcome, and sample size, affect the magnitude of bias in the regression coefficients of the skill and a covariate?

By addressing these questions, we aim to close the aforementioned gap in the methodological literature and advance socio-emotional skill assessments with regard to scoring practices. This issue is timely because scoring is an area where socio-emotional skill assessments—and indeed the assessment of any construct based on rating scales—are still lagging behind the methodological standards and best practices of cognitive skill assessments. Our ultimate goal is to help researchers as well as data producers to make informed choices about how to score, or perhaps *whether or not* to score, socio-emotional skill measures.

## 5. Methods

### 5.1. Design of the Simulation Study

We considered four factors in the design of the simulation study: Number of items, factor loadings of the item on the latent skill θ, relative strength of the relationship of the skill with the outcome, and sample size. We chose the levels of these factors to closely match typical socio-emotional skill and personality scales (see section 3). Table 4 details the factors that were manipulated in the simulation study.

**Table 4:** Design of the simulation study.

**Factors**	**Levels**	**Total number of levels**
Number of items	4, 8, 12	3
Factor loadings	All high, mixed, all low	3
Sample size	300 (small), 1,000 (large)	2
Relative strength of the relationship between the skill and the outcome	Greater than the covariate, lesser than the covariate	2
Approaches	MS, EBM, RFS, WLE, EAP, PV, and SEM	7

#### 5.1.1. Number of Items

Socio-emotional skills and personality scales use 4 to 8 items per dimension, whereas longer scale are rare (e.g., du Plessis and de Bruin, 2015; Soto and John, 2017; Soto et al., 2021). Hence, we considered 4, 8, and 12 number of items per scale to represent short, medium, and long unidimensional scales, respectively.

#### 5.1.2. Factor Loadings of the Item

We considered scales with high, mixed, and low factor loadings in our simulation study. In a scale with high factor loadings, all the items have factor loadings of either 0.7 or 0.8. In case of scale with mixed factor loadings, the items have factor loadings ranging from 0.4 to 0.9. In a scale with low factor loadings, all the items have factor loadings of either 0.4 or 0.5. Table 5 presents the scale reliability in terms of ω (McDonald, 1999; Hayes and Coutts, 2020) implied by the different combinations of number of items and factor loadings used in our study. The scale reliability ranges from 0.5 to 0.94 under different conditions.

**Table 5:** Scale reliabilities, ω, of the unidimensional skills considered in the simulation study for different number of items and the strength of the factor loadings.

**Number of items**	**Factor Loadings**		
	**High**	**Mixed**	**Low**
4	0.84	0.76	0.50
8	0.91	0.86	0.67
12	0.94	0.90	0.75

#### 5.1.3. Relative Strength of the Relationship of the Skill With the Outcome

Because incremental validity questions are so common in research on socio-emotional skills and personality, in this study we compare the efficacy of different approaches of analyzing SES items in recovering regression parameter not only of a skill but also that of a covariate. Thus, in addition to assessing bias in the regression coefficient of a skill, we also assess bias in the regression coefficient of a covariate that results when using different approaches to analyzing the skill (i.e., test scores, PV, SEM). We consider two cases: (1) the skill is more strongly correlated with the outcome variable than the covariate, and (2) the covariate is more correlated with the outcome variable than the skill.

#### 5.1.4. Sample Size

Previous studies on analyzing skills from large scale cognitive studies have mostly concentrated on large sample sizes that are typical of LSAS (see Table 2). Large samples are advantageous in that they ensure stable estimates and sufficient statistical power for most types of analysis. However, much—and probably most—research on SES or personality is based on smaller samples and are not representative or large like LSAS samples. An analysis of sample sizes in six well-regarded journals in personality psychology found that the median sample size was only 104 and hardly increased over the years (Fraley and Vazire, 2014), although it should be noted that this included both experimental designs and correlational designs (e.g., surveys); the latter typically have much larger sample sizes, and samples of 300 to 500 respondents are easy to acquire nowadays through online surveys. Certain approaches of analyzing SES items (e.g., item factor analysis with weighted least-squares [WLS] estimators) require larger samples to produce reliable and stable estimates. Hence, in this study, we will explore the effect of two levels of sample sizes: 300 and 1,000 to represent small and large samples, respectively.

### 5.2. Model Specification

As described in Figure 1, we generated data for the simulation study such that for a particular sample size, a number of items *X*_1_, …, *X*_*k*_ were observed measures of the latent variable θ representing the skill, with factor loadings λ_1_, …, λ_*k*_ depending on the different levels of the factor loading design factor. Each item had zero mean and unit variance, and the items followed a multivariate normal distribution with unidimensional confirmatory factor analytic model implied covariance. We then categorized the initially continuous items into 5 ordinal response categories, such that the resulting responses form a symmetric bell-shaped histogram.

The skill θ was correlated with a single covariate, denoted *C*. We fixed the correlation between them, ϕ, at (φ = 0.30) for all conditions. The covariate also had a zero mean and unit variance. Furthermore, there were two continuous outcome variables, *Y*_1_ and *Y*_2_. Both θ and the covariate *C* were predictors of both these outcomes. For outcome *Y*_1_, we fixed the regression coefficients such that β_1_ > γ_1_, indicating that the skill was more strongly correlated with the outcome than the covariate. For outcome *Y*_2_, we fixed the regression coefficients such that β_2_ < γ_2_, indicating that the covariate was more strongly correlated with the outcome than the skill.

In all, we generated data for 36 conditions (refer Table 4) and compared the performance of different approaches of analyzing the skill as a predictor in multiple regression. We replicated each condition 500 times. For each condition the same starting seed was used as a variance reducing method (Boomsma, 2013). R Studio (R Core Team, 2020) with *lavaan* (Rosseel, 2012) and *TAM* (Robitzsch et al., 2020) packages were used for data generation and data analyses.

### 5.3. Computing Test Scores

For each simulation condition, we computed five types of test scores that are widely used in applied research and/or discussed in the methodological literature: Standardized mean scores (SMS), regression factor scores (RFS), empirical Bayes modal (EBM) scores, weighted maximum likelihood estimates (WLE), and expected a posteriori (EAP) estimates. Below we describe the computational details of each.

#### 5.3.1. Standardized Mean Scores (SMS)

Mean scores are the simplest and most widely used type of test scores for constructs that are measured with multi-item scales that use a rating scale format (McNeish and Wolf, 2020; Lechner et al., 2021). As in much of applied research, here we will consider standardized mean scores^6^. Consider *x*_*ij*_ to be the response of respondent *i* (i = 1, …, n) on item *j* (j = 1, …, m). SMS is computed as


$$\begin{array}{l} {\hat{\theta }}_{i}^{MS}=\frac{1}{m}\overset{m}{\underset{j=1}{\sum }}x_{ij};\;i=1,\ldots ,n\\ {\hat{\theta }}_{i}^{SMS}=\frac{{\hat{\theta }}_{i}^{MS}-{\bar{\theta }}^{MS}}{\sigma_{{\hat{\theta }}^{MS}}} \end{array}$$

where ${\bar{\theta }}^{MS}$ is mean and $\sigma_{{\hat{\theta }}^{MS}}$ is the standard deviation of the mean scores.

Different from the other four types of test scores described below, SMS can be calculated directly from the item responses. More complex method require a two-step process (Rdz-Navarro, 2019): In the first step, an appropriate measurement model is estimated. In the second step, the scores are estimated for each response pattern using the model parameters from the first step. However, it is important to realize that SMS is in fact, based on rather strong assumptions about the underlying measurement model (e.g., von Davier, 2010; Beauducel and Leue, 2013; McNeish and Wolf, 2020): SMS implicitly assumes a model of “parallel tests”—a rather unrealistic assumption for socio-emotional skills and personality scales in which items almost invariably have different loadings, intercepts, and residual variances.

#### 5.3.2. Regression Factor Scores (RFS)

Another type of widely used test scores are RFS computed from classical test theory (CTT) measurement models such as confirmatory factor analysis. Skrondal and Laake (2001) noted that for explanatory variables, RFS, extracted from a factor model, tend to produce consistent estimators for all parameters. Consider the following factor model:


$$\begin{array}{l} X=\Lambda_{X}\xi +\delta \end{array}$$

where X is a response matrix with entries *X*_*ij*_ indicating the response of respondent *i* (i = 1, …, n) on item *j* (j = 1, …, m). Λ_*X*_ is the matrix of factor loadings, ξ is the vector of latent variables, and δ is the vector of errors. RFS can then be computed by regressing


$$\begin{array}{l} {\hat{\theta }}^{RFS}=\Phi \Lambda_{X}^{T}\Sigma_{X}^{-1} \end{array}$$

where ${\hat{\theta }}^{RFS}$ is the matrix of RFS for all respondents. Φ is the covariance matrix of ξ and Σ_*X*_ is the model implied covariance matrix. In this study, we used robust maximum likelihood (MLR) estimation for the parameters of the confirmatory factor analysis (CFA) model.

#### 5.3.3. Weighted Maximum Likelihood Estimator (WLE) Scores

WLE is a popular choice for computing test scores when item response theory (IRT) models such as the 2-PL model are used. WLE corrects for the bias in the asymptotic variance of the maximum likelihood estimator (MLE) (Warm, 1989). Consider *m* polytomous items j = 1, …, m. Let each of these items have *r* response categories k = 1, …, r. Let θ_*i*_ be the trait level of respondent i (i = 1, …, n) and *P*(*x*_*jk*_|θ_*i*_) be the probability of respondent with trait θ_*i*_ selecting category k on item j. The likelihood function is given as


(1) $$\begin{array}{l} L\left(x|\theta \right)=\overset{n}{\underset{i=1}{\prod }}\overset{m}{\underset{j=1}{\prod }}\overset{r}{\underset{k=1}{\prod }}{\left[P\left(x_{jk}|\theta_{i}\right)\right]}^{x_{jk}} \end{array}$$

Warm's likelihood function is defined as


$$\begin{array}{l} L^{*}\left(x|\theta \right)=f\left(\theta \right)L\left(x|\theta \right) \end{array}$$

where *f*(θ) is the square root of the test information.


$$\begin{array}{l} {\hat{\theta }}^{WLE}=\underset{\theta }{arg max}L^{*}\left(x|\theta \right) \end{array}$$

While the asymptotic variance of WLE continues to be biased, its bias is smaller than that of MLE. As MLE is theoretically unbiased, so are WLE (Rdz-Navarro, 2019). In this study, we used a 2-PL generalized partial credit model (GPCM) for the responses. We estimated the parameters using maximum likelihood estimation with Gaussian quadrature approximation.

#### 5.3.4. Expected a Posteriori (EAP) Scores

Akin to WLE, EAP is widely used for computing test scores in cognitive assessments. However, unlike WLE, EAP requires a prior distribution of θ. EAP estimate is the mean of the posterior distribution of θ, which combines information about response patterns and model parameters with a prior distribution. Shrinkage toward the population mean can be reduced by including background information in the prior distribution of θ. For a given prior distribution *g*(θ) of the respondent's ability, the posterior distribution is defined as -


$$\begin{array}{l} P\left(\theta |x\right)=\frac{L\left(x|\theta \right)g\left(\theta \right)}{P\left(x\right)};\;P\left(x\right)=\int L\left(x|\theta \right)g\left(\theta \right)d\theta \\ {\hat{\theta }}^{EAP}=E\left(\theta |x\right)=\int \theta P\left(\theta |x\right)d\theta \end{array}$$

Similar to WLE scores, we used a 2-PL GPCM with Gaussian priors for the responses in this study. We estimated the parameters using maximum likelihood with Gaussian quadrature approximation.

#### 5.3.5. Empirical Bayes Modal (EBM) Scores

In empirical Bayes estimation of θ, posterior mean of θ is obtained with the parameter estimates plugged in. EBM estimates make use of posterior mode instead of posterior mean. Posterior mode minimizes the posterior expectation of the zero-one loss function thereby reducing the misclassifications (Rabe-Hesketh et al., 2004). This makes EBM especially well suited for categorical data. Similar to EAP, background information or covariates can be included in the prior distribution to obtain better EBM estimates. Consider $P\left(\theta |x;\hat{\theta }\right)$, the conditional posterior distribution of θ given the estimated parameters


$$\begin{array}{l} {\hat{\theta }}^{EBM}=\underset{\theta }{max arg}P\left(\theta |x;\hat{\theta }\right) \end{array}$$

In this study, we used weighted least square mean and variance (WLSMV) adjusted estimators with Gaussian priors.

### 5.4. Generating Plausible Values

For each simulated dataset, we estimated a set of 10 PV per hypothetical respondent. For item response matrix *x* and ability θ, *P*(*x*|θ) represents the item response or the measurement model. Further, the prior distribution *g*(θ) is typically assumed to follow normal distribution given *c*, a vector of background or conditioning variables (Wu, 2005):


$$\begin{array}{l} g\left(\theta |c\right)∽N\left(\mu +\beta c,\sigma^{2}\right) \end{array}$$

In the PV literature and in LSAS, *g*(θ|*c*) is referred to as the “background model” or “conditioning model”.

PV are, then, generated as *m* random draws drawn from the posterior distribution *P*(θ|*x, c*), i.e. ${\hat{\theta }}_{l}^{PV}∽P\left(\theta |x,c\right)$. Subsequent analyses is performed for each ${\hat{\theta }}_{l}^{PV}$ and the final estimate if obtained by pooling all *m* estimates using missing value imputation methodology (Wu, 2005; von Davier et al., 2009).

For generating PV, we used a 2-PL generalized partial credit model (GPCM) as response model with marginal maximum likelihood (MML) estimation using quasi Monte Carlo integration for each condition. The covariate, *C* and the two outcome variables—*Y*_1_ and *Y*_2_ (from Figure 1) were used as background variables in the population model for PV. We used the *TAM* package (Robitzsch et al., 2020) to generate PV and the *miceadds* package (Robitzsch and Grund, 2021) to pool the results of the regressions with PV as predictor.

### 5.5. Structural Equation Model (SEM)

We fit a SEM with a CFA measurement model (as shown in Figure 1) to each simulated dataset. We fixed the variance of the latent skill θ to unity and freely estimated the factor loadings of all items. We included the correlation between the skill and the covariate in the structural model. To estimate the SEM, we used the R package *lavaan* (Rosseel, 2012) with a robust maximum likelihood (MLR) estimator.

### 5.6. Estimating Bias in Regression Coefficients

The main goal of this study was to examine how the different approaches of analyzing socio-emotional skills (the five types of test scores, SEM, and PV) recover regression coefficients of both the skill and the covariate in multiple regression. Hence, the outcomes of interest in this simulation study are: (1) the percent bias in the regression coefficient of the skill, and (2) the percent bias in the regression coefficient of the covariate. We calculate percent bias in the regression coefficients of both the skill and the covariate for each replication under each condition as:


$$\begin{array}{l} \%Bias=100\times \frac{\hat{\beta }-\beta }{\beta } \end{array}$$

where β is the population value and $\hat{\beta }$ is the estimated value of the regression coefficient.

There is no universal answer as to what amount of bias is acceptable, mild or severe. In previous simulations, percent absolute relative bias in regression coefficient was often deemed acceptable if it was below 10% (Hoogland, 1999; Poon and Wang, 2010; Leite, 2017). However, this is merely a rule of thumb. Depending on the research context, even an absolute relative bias of less than 10% can be problematic, especially in cases involving high-stakes decisions. In other cases such as exploratory low-stakes research, absolute relative bias up to 15% might sometimes be deemed acceptable. As a rough and tentative guideline based on prior work, we interpreted bias of less than 5% as “ignorable,” bias of between 5 and 10% as “likely unproblematic,” and bias of more than 10% as “likely problematic.”

We also obtained correlations among the 5 types of test scores and the two outcome variables for each replication under each condition. We then pooled these correlations across the 500 replications for each condition and then further pooled them across all conditions to obtain a single estimate for each correlation.

## 6. Results

### 6.1. Correlations Between Skill Scores

**Figure 2:**
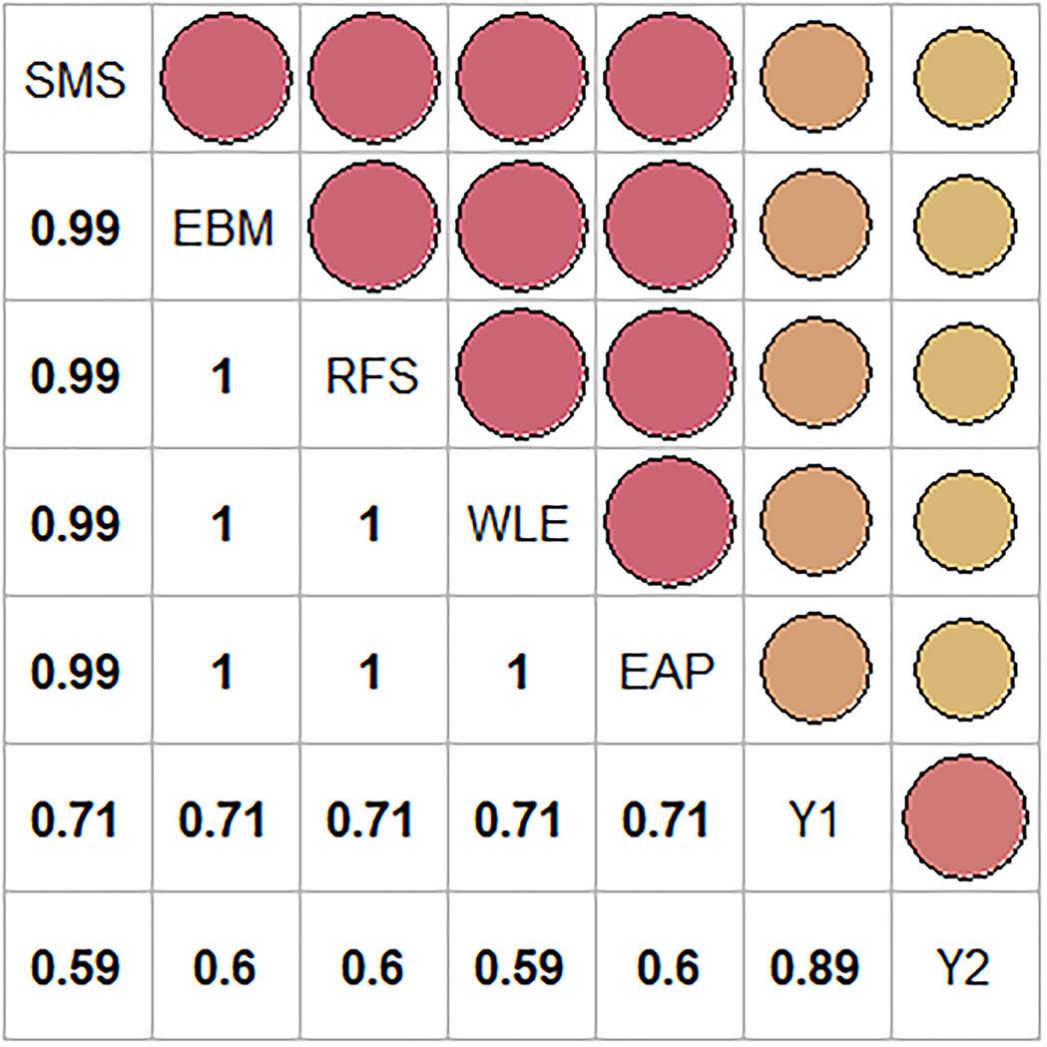
Correlation plot of the five types of test scores-SMS, EBM, RFS, WLE, and EAP, and the two outcomes - *Y*_1_ and *Y*_2_. The size and color of the dots in the plot represent the strength of the correlation. Bigger the dots, higher the correlation.

Figure 2 presents the correlation between the five types of test scores (SMS, EBM, RFS, WLE, and EAP). The correlations were extremely high, approaching unity. The correlations of these test scores with the two outcomes were almost identical across the different types of test scores.

These correlations would seem to suggest that all types of test scores yield highly similar results. However, this does not necessarily imply that the scoring methods are created equal or that they can be used interchangeably when it comes to bias in regression coefficients, because the regression coefficient also depends on the ratio of the standard deviation of the outcome to the standard deviation of the scores. As this ratio is different for different scores, the regression coefficients are bound to be different for the different types of test scores.

### 6.2. Bias in the Regression Coefficient of the Skill

#### 6.2.1. Performance of the Different Approaches With Regard to Percentage Bias

**Figure 3:**
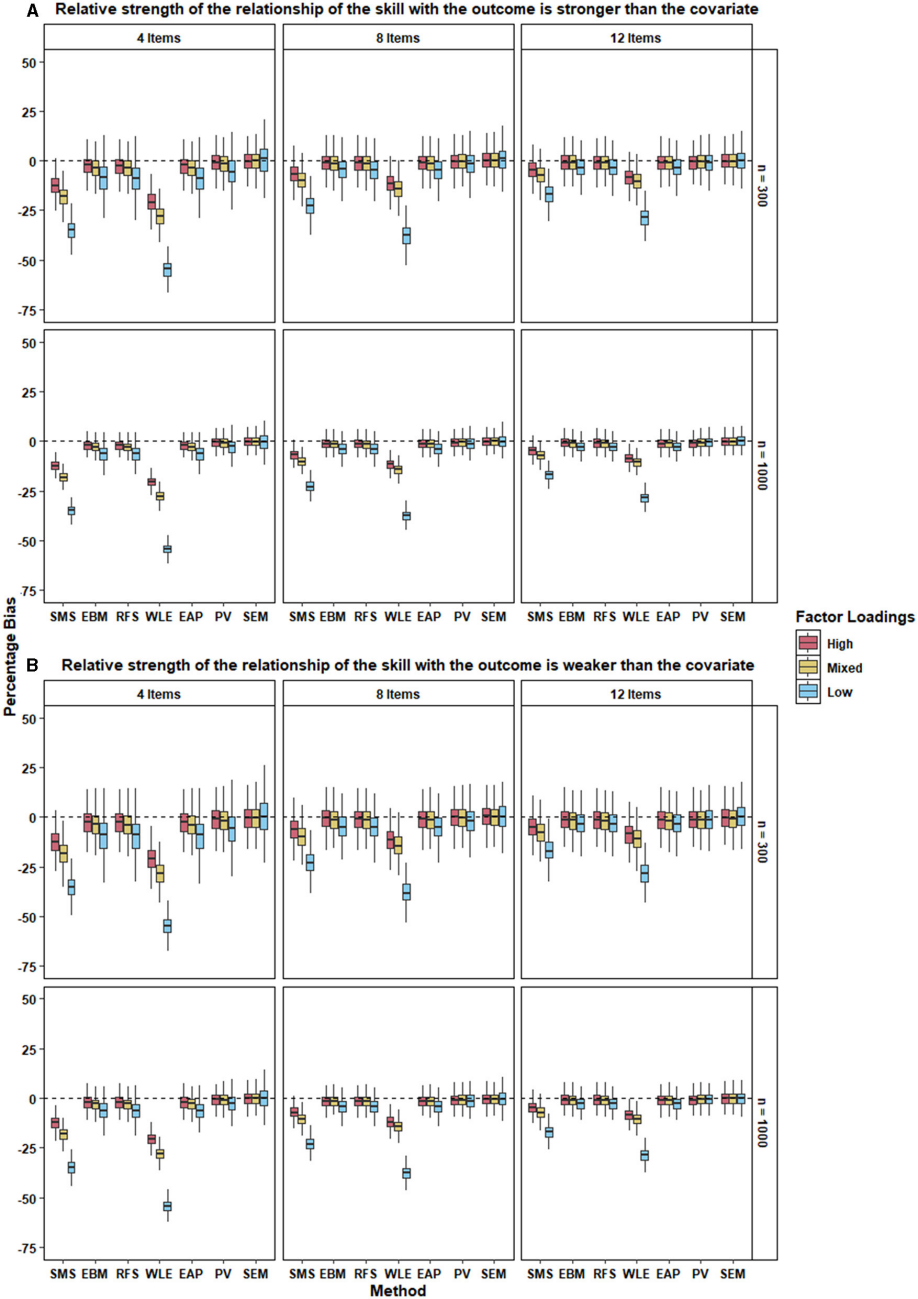
The two panels, **(A,B)**, present the boxplot of percentage bias in the regression coefficient of the skill for the different approaches under each condition when the relative strength of the relationship between the skill with the outcome is stronger and weaker respectively, than the covariate. 500 replications of each condition were used to create the boxplot.

Figure 3 and Table 6 show the performance of the different approaches in terms of percentage bias. SEM performed the best in terms of recovering the regression coefficient of the skill. SEM had the lowest mean percent bias (<1%) across all conditions, meaning that it almost perfectly recovered the population regression coefficients. Mean percent bias of PV across all conditions was <3%. Hence, PV performed almost as well as SEM.

**Table 6:** Mean percentage bias of regression coefficient of the skill for different approaches for each condition.

**RSRO**	**SS**	**FL**	**Items**	**SMS**	**EBM**	**RFS**	**WLE**	**EAP**	**PV**	**SEM**
Stronger than the covariate	300	High	4	–12.28	–2.33	–2.49	–20.62	–2.43	–0.74	–0.17
			8	–6.53	–1.10	–1.25	–11.59	–1.18	–0.46	0.02
			12	–4.61	–0.90	–1.03	–8.37	–1.14	–0.87	–0.20
		Mixed	4	–18.11	–3.53	–3.69	–28.02	–3.66	–1.47	–0.24
			8	–9.96	–1.33	–1.49	–14.12	–1.41	–0.41	0.10
			12	–7.23	–1.13	–1.26	–10.44	–1.18	–0.66	–0.17
		Low	4	–34.97	–8.76	–9.04	–54.61	–9.04	–5.36	0.85
			8	–22.72	–4.66	–4.83	–37.67	–4.84	–1.66	0.39
			12	–16.75	–3.31	–3.44	–28.57	–3.44	–1.03	0.24
	1,000	High	4	–12.27	–2.07	–2.12	–20.43	–2.04	–0.54	–0.09
			8	–6.74	–1.19	–1.23	–11.67	–1.17	–0.60	–0.14
			12	–4.68	–0.86	–0.90	-8.49	–1.12	–0.93	–0.15
		Mixed	4	–18.12	–2.85	–2.89	–27.77	–2.85	–0.74	–0.09
			8	–10.23	–1.40	–1.44	–14.16	–1.35	–0.52	–0.12
			12	–7.22	–1.05	–1.09	–10.45	–1.06	–0.61	–0.12
		Low	4	–34.99	–6.31	–6.37	–54.43	–6.37	–2.57	–0.07
			8	–22.80	–3.84	–3.88	–37.56	–3.88	–0.98	–0.01
			12	–16.88	–2.68	–2.71	–28.63	–2.71	–0.42	0.19
Weaker than the covariate	300	High	4	–12.49	–2.57	–2.73	-20.82	–2.67	–1.04	–0.41
			8	–6.30	–0.84	–0.98	–11.35	–0.91	–0.18	0.28
			12	–4.79	–1.08	–1.21	–8.53	–1.31	–1.06	–0.38
		Mixed	4	–18.22	–3.67	–3.83	–28.13	–3.81	–1.68	–0.40
			8	–9.71	–1.08	–1.24	–13.90	–1.16	–0.15	0.35
			12	–7.59	–1.54	–1.68	–10.82	–1.60	–1.10	–0.59
		Low	4	–35.06	–8.99	–9.27	–54.74	–9.28	–5.61	0.74
			8	–22.75	–4.69	–4.86	–37.69	–4.86	–1.69	0.37
			12	–16.64	–3.10	–3.23	-28.41	–3.23	–0.81	0.43
	1,000	High	4	–12.34	–2.17	–2.22	–20.50	–2.13	–0.65	–0.19
			8	–6.92	–1.37	–1.41	–11.86	–1.37	–0.80	–0.33
			12	–4.66	–0.84	–0.88	–8.47	–1.10	–0.90	–0.12
		Mixed	4	–18.16	–2.94	–2.99	–27.83	–2.94	–0.85	–0.19
			8	–10.42	–1.59	–1.64	–14.35	–1.55	–0.74	–0.32
			12	–7.16	–1.00	-1.04	–10.42	–1.02	–0.57	–0.07
		Low	4	–34.87	–6.14	–6.20	–54.35	–6.20	–2.39	0.11
			8	–23.00	–4.08	–4.13	–37.73	–4.13	–1.24	–0.27
			12	–17.00	–2.83	–2.86	–28.73	–2.86	–0.58	0.05

*The mean percentage bias was calculated by aggregating percentage bias across 500 replications for each condition*.

*Note: RSRO: Relative strength of the skill with the outcome, SS: Sample size, FL: Factor loadings*.

As expected, all 5 types of test scores produced higher bias than SEM and PV. Importantly, despite their strong intercorrelations, the performance of the different test scores varied markedly across the conditions. EBM, RFS, and EAP performed equivalently and relatively well with mean percent bias <10% across all conditions. However, their performance was clearly worse than that of PV and SEM. SMS performed poorly with mean percent bias ranging from 5% to up to 35% under different conditions. WLE had the worst performance of all approaches with the mean percent bias ranging from 8 – 55% for different conditions.

#### 6.2.2. Effects of Experimental Factors on Percentage Bias

Next, we probed how the different factors in our simulation affected the amount of bias in the regression coefficient. For all the different approaches, percent bias decreased when the scale comprised a larger number of items. This trend held for all levels of factor loadings, relative strength of the relationship of the skill with outcome, and the sample size.

Percent bias was also lower for all methods when the factor loadings were high (i.e., when scale reliability was higher; see Table 5). Percentage bias was slightly higher for mixed factor loadings and the highest for low factor loadings. This trend was evident across the different levels of number of items, relative strength of the relationship of the skill with outcome, and the sample size.

As evident from and Figure 3, the relative strength of the relationship between the skill and the outcome did not affect the bias in the regression coefficients of the skill. This was true for all approaches under all conditions. Similarly, sample size did not alter the performance of different approaches under different conditions. However, variability in the percentage bias of the approaches was larger for small sample size compared with that of the large sample size for all conditions.

### 6.3. Bias in the Regression Coefficient of the Covariate

#### 6.3.1. Performance of the Different Approaches With Regard to Percentage Bias

**Figure 4:**
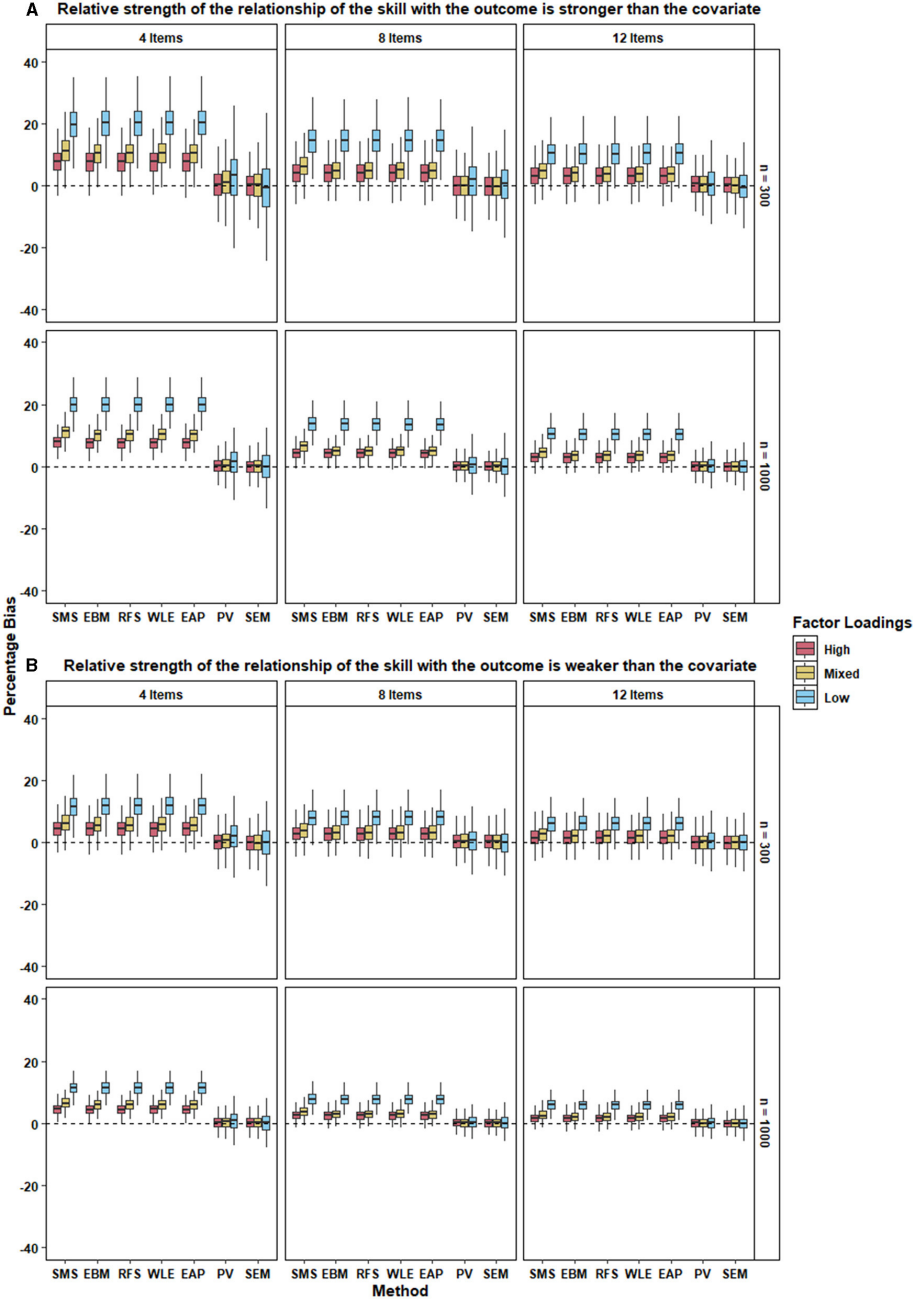
The two panels, **(A,B)**, present the boxplot of percentage bias in the regression coefficient of the covariate for the different approaches under each condition when the relative strength of the relationship between the skill with the outcome is stronger and weaker respectively, than the covariate. 500 replications of each condition were used to create the boxplot.

How does the way in which the different approaches account (or fail to account) for measurement error in the skill affect the bias in the regression coefficient of a covariate? From Figure 4, it is clear that SEM performed best in terms of recovering the regression coefficient of the covariate across all conditions. PV performed on par with SEM, with a mean percent bias <3% for all conditions (see Table 7).

**Table 7:** Mean percentage bias of regression coefficient of the covariate for different approaches for each condition.

**RSRO**	**SS**	**FL**	**Items**	**SMS**	**EBM**	**RFS**	**WLE**	**EAP**	**PV**	**SEM**
Stronger than the covariate	300	High	4	7.68	7.57	7.57	7.64	7.56	0.35	–0.01
			8	4.07	3.99	3.99	4.02	3.97	–0.08	–0.30
			12	3.21	3.17	3.17	3.17	3.17	0.52	0.27
		Mixed	4	11.19	10.40	10.40	10.53	10.40	1.12	0.28
			8	6.25	4.88	4.88	4.98	4.86	0.04	–0.28
			12	4.79	3.78	3.79	3.80	3.75	0.34	0.08
		Low	4	19.69	20.12	20.14	20.17	20.15	2.74	–1.14
			8	14.40	14.55	14.56	14.57	14.56	1.62	0.35
			12	10.33	10.41	10.41	10.42	10.41	0.60	–0.29
	1,000	High	4	7.84	7.60	7.60	7.65	7.58	0.22	0.06
			8	4.33	4.19	4.19	4.23	4.17	0.21	0.05
			12	2.97	2.88	2.88	2.88	2.86	0.16	0.00
		Mixed	4	11.27	10.16	10.16	10.28	10.16	0.33	0.04
			8	6.55	5.00	5.00	5.15	4.99	0.21	0.04
			12	4.55	3.62	3.62	3.66	3.59	0.11	–0.03
		Low	4	20.01	19.96	19.97	20.00	19.97	1.31	–0.08
			8	13.79	13.69	13.69	13.70	13.69	0.51	0.03
			12	10.61	10.50	10.50	10.50	10.50	0.33	0.03
Weaker than the covariate	300	High	4	4.24	4.18	4.18	4.22	4.18	0.10	–0.11
			8	2.73	2.68	2.68	2.70	2.67	0.36	0.24
			12	1.62	1.59	1.59	1.59	1.59	0.09	–0.05
		Mixed	4	6.17	5.72	5.72	5.80	5.72	0.47	–0.02
			8	3.91	3.13	3.14	3.19	3.12	0.38	0.20
			12	2.62	2.06	2.06	2.07	2.04	0.11	–0.04
		Low	4	11.45	11.71	11.72	11.73	11.72	1.85	–0.40
			8	7.79	7.88	7.89	7.89	7.89	0.54	–0.19
			12	6.01	6.04	6.04	6.05	6.04	0.45	–0.04
	1,000	High	4	4.58	4.44	4.44	4.47	4.43	0.26	0.16
			8	2.53	2.45	2.45	2.47	2.44	0.19	0.10
			12	1.69	1.64	1.64	1.64	1.63	0.09	0.00
		Mixed	4	6.55	5.93	5.93	6.00	5.93	0.36	0.19
			8	3.82	2.94	2.94	3.03	2.93	0.23	0.13
			12	2.57	2.05	2.05	2.07	2.03	0.05	–0.03
		Low	4	11.44	11.41	11.41	11.43	11.42	0.80	0.01
			8	7.86	7.80	7.80	7.81	7.80	0.34	0.06
			12	5.99	5.92	5.92	5.93	5.92	0.16	–0.02

*The mean percentage bias was calculated by aggregating percentage bias across 500 replications for each condition*.

*Note: RSRO: Relative strength of the skill with the outcome, SS: Sample size, FL: Factor loadings*.

All types of test scores performed worse than PV and SEM, but were similar to each other, with their mean percent bias ranging from 2–20% under different conditions. It is interesting to note that SMS performed no worse than more sophisticated types of test scores in recovering the regression coefficient of the covariate.

#### 6.3.2. Effects of Experimental Factors on Percentage Bias

Similar to the recovery of the regression coefficient of the skills, the percent bias in the regression coefficient of the covariate was smaller when the scale comprised a larger number of items. This trend was evident for all the approaches—regardless of the level of factor loadings, relative strength of the relationship of the skills with outcome, and the sample size.

Percent bias was also lower for all approaches when the factor loadings were all high (i.e., when scale reliability was high; see Table 5). It was only slightly higher when factor loadings were mixed and the highest when the factor loadings were low. We also observed that the variability in the percentage bias increased as the strength of the factor loadings decreased. This trend was observed across the different levels of number of items, relative strength of the relationship of the skills with outcome, and the sample size.

As evident from Figure 4, the percent bias of a given approach in case where the relative strength of the relationship of the skill with the outcome is higher than the covariate, was comparable to that where the relative strength is weaker. This was true for all approaches under all conditions. The only notable exception to this pattern was the bias in the various types of test scores in the condition with low factor loadings; this bias was smaller when the relative strength of the relationship of the skill with the outcome was lower (Figure 4B) compare to when it was higher (Figure 4A).

Again, sample size did not seem to affect the performance of different approaches under different conditions. However, as seen earlier, variability in percent bias of the methods was larger for small sample size across all conditions.

### 6.4. Additional Analyses: Bias in Standardized Regression Coefficient

As mentioned earlier, we used the standardized mean score in the regression to ensure that the mean score was meaningful with regard to the population metric of the skill (i.e., zero mean and unit variance) and to allow for meaningful comparisons with other approaches. We did not standardize the other test scores (or PV) because they are already in the population metric of the skill that we specified in the simulation (i.e., zero mean and unit variance).

As it is a common practice in studies on socio-emotional skills or personality to report standardized regression coefficients in order to interpret relationships with educational or life outcomes (Richards, 1982; Courville and Thompson, 2001), we also obtained standardized regression coefficients for both the skill and the covariate for the remaining test scores (EBM, RFS, WLE, EAP). We provide tables with the mean percent bias in the standardized regression coefficients of skill and covariate in the Tables A1, A2 in Appendix, respectively.

These additional analyses showed that the performance of EBM, RFS, and EAP, though comparable with each other, worsened in terms of percent bias when using standardized instead of unstandardized regression coefficients. The mean percent bias for these three test scores ranged from 5 – 37%. Contrariwise, standardization of WLE scores drastically improved their performance compared with its unstandardized regression coefficient (compare Table 6 with Table A1 in Appendix). Performance of the four test scores—EBM, RFS, WLE, and EAP—was similar across all conditions. Furthermore, standardization of the test scores did not change the percent bias in the regression coefficient of the covariate (Table A2 in Appendix). It is identical to bias in case of unstandardized regression coefficients of the four test scores (Table 7).

## 7. Discussion

In this simulation study, we compared the performance of three principal approaches (test or scale scores, SEM, and PV) for analyzing socio-emotional skills scales in regression analyses where the skill is a predictor. Although our study was motivated by the growing number of studies on socio-emotional skills, our findings apply equally to measures of personality traits, motivation, goals, attitudes—indeed any multi-item scale that seeks to measure a unidimensional latent construct with relatively few (i.e., 4–12) items using a polytomous (rating scale) response format.

In terms of recovering the regression coefficient of the skill, some test scores (EBM, EAP, and RFS) mostly performed adequately even for scales with fewer items and mixed or low factor loadings. These test scores produced only mild bias in the regression coefficient for the skills that is likely to be inconsequential for research findings. By contrast, the two other types of test scores (SMS and the WLE) often performed poorly, resulting in bias that far exceeds the threshold of what is commonly seen as ignorable or acceptable. Notably, the very high correlations among different types of test scores did not translate into similar magnitudes of percentage bias in the regression coefficients of the skill. Different types of test scores cannot and should not be used interchangeably, even though they may be highly correlated. Moreover, as additional analyses showed, the performance of test scores varies widely depending on whether unstandardized scores (as in our main analyses) or standardized scores (as in our additional analyses) are used. The superior performance of SEM and PV was noteworthy under all conditions: Both methods yielded bias that was small enough to be safely ignored in most applied research scenarios.

In terms of recovering the regression coefficient of a covariate, test scores did not perform satisfactorily. Especially for scales with fewer items and mixed or low factor loadings, bias often reached levels that are likely problematic. This indicates that whereas using test scores such as RFS, EAP, and EBM results in negligible bias in recovery of regression coefficient of the skill, using test scores can still entail considerable bias in recovering the regression coefficient of covariates, potentially leading to erroneous research findings. Contrariwise, the performance of PV and SEM was excellent under all conditions. As one would expect, both methods almost completely eliminated bias in the regression coefficient of the covariate under all conditions.

Our results expand previous simulation studies on scoring, SEM, and PV. As previous studies mostly hail from the realm of cognitive assessments and mirror the conditions that are typical of those assessments (see Table 2), it is instructive to compare the findings of these studies with our own. Similar to previous simulations (see Table 3), we found that PV performed exceptionally well and under most conditions comparable to SEM. We also saw that some of the test scores (RFS, EBM, and EAP) performed similar to each other in most cases. Increase in number of items improved the performance of all approaches. Similar to these earlier studies, sample size had no bearing on the differences in the percent bias for the different methods in our simulation. Distinct from some previous simulations, PV performed well even for small sample sizes and low factor loadings. Even though the some of the test scores such as RFS, EBM, and EAP had higher bias than PV, this bias was negligible for most conditions in terms of recovery of regression coefficient of the skill. Although WLE performed better with increase in the number of items, its bias was still likely problematic and in certain conditions it was worse than SMS. In sum, our results partly align with those of prior simulation studies, especially in highlighting PV and SEM as effective in removing bias from regression coefficients, but partly deviate from them and are more nuanced. Moreover, none of the previous simulation compared different types of test scores to SEM and PV, as we did in our study.

### 7.1. Limitations and Directions for Future Research

Like all simulation studies, our study has limitations in the form of generalizability. Even though we designed our simulations to closely match the real data scenarios in studies on socio-emotional skills, there are several issues that we could not cover here: missing data, which complicates usage of test scores but not SEM or PV (von Davier et al., 2009; Braun and von Davier, 2017), small sample size issues, and non-classical measurement error, which determines the form of bias (attenuation or inflation; Fuller, 2006; Schofield, 2015). We also did not investigate different response formats and multidimensional skills. Often in socio-emotional or personality skills assessments, it is common for the skills to be correlated with each other, and skills are analyzed simultaneously as multi-dimensional inventories (e.g., Soto and John, 2017; Soto et al., 2021). Future research can focus on examining the performance of the three approaches in the case of missing data, non-classical measurement error, and multi-dimensional scales.

### 7.2. Practical Implications and Recommendations

Findings from our simulation beg the question: “To score or not to score?.” We demonstrate that using test scores (fallible point estimates of individuals' skills) can result in considerable bias in both the regression coefficient for the skill that is modeled as a predictor (which is typically underestimated) and in the regression coefficient for a covariate (which is typically overestimated). This bias occurs in many conditions typical of socio-emotional skill assessments. Moreover, it occurs especially with simple (i.e., SMS) but also with more advanced (e.g., WLE) types of test scores.

The situation is thus reminiscent of cognitive skill assessments, where the use of test scores has now been discouraged in favor of PV methodology (Wu, 2005; von Davier et al., 2009; Laukaityte and Wiberg, 2018). Given how crucial scale reliability turned out for the magnitude of bias in our simulations, it can be argued that recommendations against using test scores apply with even greater force to socio-emotional skill assessments. This is because these assessments often involve shorter scales (e.g., 4–6 items) with comparatively lower reliabilities, resulting in greater bias in regression coefficients of both the skill and the covariates.

In view of this, our recommendations are threefold. First, applied researchers who analyze data from socio-emotional skill assessments should employ SEM or PV instead of using fallible test scores. This is because SEM explicitly models measurement error and PV implicitly corrects for the uncertainty about the true skill score of each respondent. Both approaches will keep bias in regression coefficient within acceptable range in most circumstances, provided that the measurement model is correctly specified.

Second, if using test scores is unavoidable, researchers should choose the type of test scores consciously and exert caution in interpreting results. There may be cases in which computing test scores is necessary. For example, if the secondary analyst intends to conduct analyses that are difficult to implement through SEM or PV framework, such as using complex survey weights (e.g., replicate weights) in analyses, fitting generalized additive models, or LOESS curve estimation, then test scores may be needed. In such cases, researchers should refrain from using the mean scores. Although mean or sum scores are still the most widely used scale scores, easy to understand, and readily interpreted, they perform sub-optimally as predictors in regression models, and worse than most of the IRT/CFA model-based scores. As we saw, high correlations among different test scores does not imply that they can be used interchangeably. Hence, researchers should prefer EBM and EAP, which lead to smaller bias. Although this is rarely implemented, EBM and EAP also allow for inclusion of covariates in the prior distribution, which improves precision (Monseur and Adams, 2009; Laukaityte and Wiberg, 2018). EAP also deals reasonably well with missing data, regardless of whether the missingness was planned or unplanned (Sengewald et al., 2018). Even when using EBM or EAP, researchers should be cautious while drawing inferences from regression analyses in which these test scores have been used in lieu of latent skills. In cases where test scores are to be reported back to respondents, SEM and PV methodologies cannot be used and researchers should provide EAP or EBM scores.

Third, data-producing organizations that curate socio-emotional skill assessments should enable secondary users of the data to use both of the approaches that account for measurement error. That is, the disseminated data should ideally include a set of PV estimated from an extensive background model that will achieve congeniality across many analysis scenarios, as is typical for cognitive assessments. Moreover, the data should include all item-level data, such that secondary analysts can estimate SEM on the original data. For data-producing organizations, PV and SEM have another advantage: In contrast to simple test scores, they can be readily applied to data from planned missingness (or “incomplete block”) designs in which each respondent answers only a subset of the total set of assessment items.

In our view, currently, PV stand out as the best option as they account for measurement error (and can incorporate information from background variables) but do not require knowledge of SEM or specialized software. Instead, all that is required is a basic understanding and implementation of multiple imputation methodology. Otherwise, the workflow for PV-based analyses is much the same as that of any other analysis with observed variables. Moreover, in contrast to SEM, PV-based analyses fulfill what Lechner et al. (2021) termed the immutability criterion—once estimated, PV do not change depending on the subsample chosen, variables included in the model, or the estimator used by the secondary analyst. This is advantageous as it will lead to higher comparability across different analyses setups and analysts, facilitating cumulative evidence on the predictive power of socio-emotional skills for life outcomes.

In sum, we hope that our findings will encourage researchers and data producers engaged in the study of socio-emotional skills, personality traits, and related constructs to embrace SEM and especially PV methodology going forward. We submit that PV should not be reserved only for cognitive assessments in LSAS. Instead, they should also be applied to socio-emotional and personality assessments. This will help minimize bias in findings on the (incremental) predictive power of such constructs for life outcomes.

## Data Availability Statement

The raw data supporting the conclusions of this article will be made available by the authors, without undue reservation.

## Author Contributions

NB: conceptualization, methodology, formal analysis, visualization, writing—original draft, and writing—revision. CL: funding acquisition, conceptualization, methodology, supervision, writing—original draft, and writing—revision. Both authors contributed to the article and approved the submitted version.

## Funding

This research was partly supported by an internal GESIS grant (Data quality indicators for multi-item scales) awarded to CL.

## Conflict of Interest

The authors declare that the research was conducted in the absence of any commercial or financial relationships that could be construed as a potential conflict of interest.

## Publisher's Note

All claims expressed in this article are solely those of the authors and do not necessarily represent those of their affiliated organizations, or those of the publisher, the editors and the reviewers. Any product that may be evaluated in this article, or claim that may be made by its manufacturer, is not guaranteed or endorsed by the publisher.

## Footnotes

^1^These umbrella terms are used rather loosely in literature, and the distinction between socio-emotional skills and personality traits is not always clear-cut. Increasingly, the Big Five domains are emerging as the dominant framework for organizing socio-emotional skill domains and for locating both new and existing scales in an established construct space. In the following, we will mostly use the term “socio-emotional skills” for simplicity yet we note that our study applies equally to personality traits and related constructs assessed through rating scales. and related constructs such as personality traits and motivation.

^2^Although conceiving of skills and traits as reflective latent variables in order to control for measurement error is common practice in psychology and neighboring disciplines, it is important to note that this practice is not without its pitfalls (e.g., Rhemthulla et al., 2020). Moreover, please note that using latent variables is not the only option for including skills or traits as predictors in regression. Researchers may also consider methods that use all single test items as predictors, such as LASSO regression or multilevel models with partial pooling (e.g., Gelman et al., 2012).

^3^The same applies to the zero-order correlation between the skill and the outcome. By contrast, the unstandardized regression coefficient (though not the standardized regression coefficient) is unbiased when the skill is an outcome instead of a predictor, even if the skill contains measurement error (Hyslop and Imbens, 2001).

^4^The first step in analyzing such multi-item skill scales almost always consists of fitting a latent measurement model, such as a confirmatory factor analysis (CFA) or graded response model (GRM). For simplicity, we assume throughout this article that this measurement model is unidimensional and correctly specified. Further, we also do not consider complications introduced by missing data that stems from respondents not completing or refusing to answer some test items.

^5^A potential downside of PV is that standard errors increase compared to test scores because of the additional uncertainty introduced by the imputation. However, with large sample sizes and a large number of PV, this is unlikely to be a major problem. In our view, unbiasedness is arguably more important than precision in the present context.

^6^In practical applications, standardization has both advantages (e.g., standard deviations are a readily interpretable metric) and disadvantages (e.g., the original metric of the response scale is lost). For our present study, the standardized mean score was the method of choice because it allowed us to interpret the scores in the population metrics that we assigned to the skill in our simulations (i.e., zero mean and unit variance; see section 5). This will also aid in fair comparison with other approaches that follow the same metric.

## Appendix

**Table A1:** Mean percentage bias of regression coefficient of the skill for standardized test scores apart from SMS.

**RSRO**	**SS**	**FL**	**Items**	**Std. EBM**	**Std. RFS**	**Std. WLE**	**Std. EAP**
Stronger than the covariate	300	High	4	-12.08	-12.07	-12.19	-12.06
			8	-6.39	-6.39	-6.44	-6.36
			12	-4.50	-4.50	-4.53	-4.50
		Mixed	4	-16.59	-16.59	-16.82	-16.58
			8	-7.67	-7.67	-7.90	-7.65
			12	-5.73	-5.72	-5.81	-5.69
		Low	4	-35.87	-35.91	-35.98	-35.92
			8	-22.95	-22.96	-22.98	-22.96
			12	-16.85	-16.86	-16.88	-16.87
	1,000	High	4	-11.90	-11.90	-12.00	-11.88
			8	-6.53	-6.53	-6.56	-6.49
			12	-4.53	-4.53	-4.54	-4.51
		Mixed	4	-16.23	-16.23	-16.42	-16.22
			8	-7.80	-7.80	-7.99	-7.76
			12	-5.72	-5.72	-5.78	-5.68
		Low	4	-34.89	-34.89	-34.95	-34.89
			8	-22.58	-22.58	-22.60	-22.58
			12	-16.68	-16.68	-16.69	-16.68
Weaker than the covariate	300	High	4	-12.29	-12.28	-12.40	-12.27
			8	-6.14	-6.14	-6.19	-6.11
			12	-4.68	-4.68	-4.70	-4.66
		Mixed	4	-16.71	-16.71	-16.95	-16.71
			8	-7.44	-7.44	-7.67	-7.41
			12	-6.12	-6.12	-6.21	-6.09
		Low	4	-36.04	-36.09	-36.15	-36.09
			8	-22.97	-22.98	-23.00	-22.98
			12	-16.67	-16.68	-16.70	-16.68
	1,000	High	4	-11.99	-11.99	-12.07	-11.97
			8	-6.70	-6.70	-6.76	-6.67
			12	-4.51	-4.51	-4.52	-4.49
		Mixed	4	-16.31	-16.31	-16.49	-16.30
			8	-7.98	-7.98	-8.19	-7.94
			12	-5.67	-5.67	-5.74	-5.63
		Low	4	-34.76	-34.77	-34.83	-34.77
			8	-22.78	-22.78	-22.80	-22.78
			12	-16.80	-16.80	-16.81	-16.80

*The mean percentage bias was calculated by aggregating percentage bias across 500 replications for each condition*.

*Note: RSRO: Relative strength of the skill with the outcome, SS: Sample size, FL: Factor loadings*.

*Mean percentage bias of regression coefficient of the covariate for standardized test scores apart from SMS*.

**Table A2:** Mean percentage bias of regression coefficient of the covariate for standardized test scores apart from SMS.

**RSRO**	**SS**	**FL**	**Items**	**Std. EBM**	**Std. RFS**	**Std. WLE**	**Std. EAP**
Stronger than the covariate	300	High	4	7.57	7.57	7.64	7.56
			8	3.99	3.99	4.02	3.97
			12	3.17	3.17	3.17	3.17
		Mixed	4	10.40	10.40	10.53	10.40
			8	4.88	4.88	4.98	4.86
			12	3.78	3.79	3.80	3.75
		Low	4	20.12	20.14	20.17	20.15
			8	14.55	14.56	14.57	14.56
			12	10.41	10.41	10.42	10.41
	1,000	High	4	7.60	7.60	7.65	7.58
			8	4.19	4.19	4.23	4.17
			12	2.88	2.88	2.88	2.86
		Mixed	4	10.16	10.16	10.28	10.16
			8	5.00	5.00	5.15	4.99
			12	3.62	3.62	3.66	3.59
		Low	4	19.96	19.97	20.00	19.97
			8	13.69	13.69	13.70	13.69
			12	10.50	10.50	10.50	10.50
Weaker than the covariate	300	High	4	4.18	4.18	4.22	4.18
			8	2.68	2.68	2.70	2.67
			12	1.59	1.59	1.59	1.59
		Mixed	4	5.72	5.72	5.80	5.72
			8	3.13	3.14	3.19	3.12
			12	2.06	2.06	2.07	2.04
		Low	4	11.71	11.72	11.73	11.72
			8	7.88	7.89	7.89	7.89
			12	6.04	6.04	6.05	6.04
	1,000	High	4	4.44	4.44	4.47	4.43
			8	2.45	2.45	2.47	2.44
			12	1.64	1.64	1.64	1.63
		Mixed	4	5.93	5.93	6.00	5.93
			8	2.94	2.94	3.03	2.93
			12	2.05	2.05	2.07	2.03
		Low	4	11.41	11.41	11.43	11.42
			8	7.80	7.80	7.81	7.80
			12	5.92	5.92	5.93	5.92

*The mean percentage bias was calculated by aggregating percentage bias across 500 replications for each condition*.

*Note: RSRO: Relative strength of the skill with the outcome, SS: Sample size, FL: Factor loadings*.

## References

[B1] Abrahams L. Pancorbo G. Primi R. Santos D. Kyllonen P. John O.. (2019). Social-emotional skill assessment in children and adolescents: advances and challenges in personality, clinical, and educational contexts. Psychol. Assess. 31, 460–473. 10.1037/pas0000591 30869960

[B2] Aiken L. West S. (1991). Multiple Regression: Testing and Interpreting Interactions. Newbury Park, CA: Sage.

[B3] Allen J. Belfi B. Borghans L. (2020). Is there a rise in the importance of socioemotional skills in the labor market? Evidence from a trend study among college graduates. Front. Psychol. 11:1710. 10.3389/fpsyg.2020.01710 32793059 PMC7392119

[B4] Aßmann C. Carstensen C. H. Gaasch C. Pohl S. (2014). Estimation of Plausible Values Using Background Variables With Missing Values: A Data Augmented MCMC Approach (NEPS Working Paper No. 38). Bamberg: Leibniz-Institute for Educational Trajectories Bamberg, National Educational Panel Study.

[B5] Beauducel A. Leue A. (2013). Unit-weighted scales imply models that should be tested! Pract. Assess. Res. Evaluat. 18:1. 10.7275/y3cg-xv71

[B6] Bergner S. (2020). Being smart is not enough: personality traits and vocational interests incrementally predict intention, status and success of leaders and entrepreneurs beyond cognitive ability. Front. Psychol. 11:204. 10.3389/fpsyg.2020.00204 32132952 PMC7040201

[B7] Bibby Y. (2020). Plausible Values: How Many for Plausible Results? (Doctoral dissertation). University of Melbourne.

[B8] Boomsma A. (2013). Reporting monte carlo studies in structural equation modeling. Struct. Equat. Model. Multidisciplinary J. 20, 518–540. 10.1080/10705511.2013.797839

[B9] Borgatto A. F. Azevedo C. Pinheiro A. Andrade D. (2015). Comparison of ability estimation methods using IRT for tests with different degrees of difficulty. Commun. Stat. Simul. Comput. 44, 474–488. 10.1080/03610918.2013.781630

[B10] Braun H. von Davier M. (2017). The use of test scores from large-scale assessment surveys: psychometric and statistical considerations. Large-scale Assess Educ. 5:17. 10.1186/s40536-017-0050-x

[B11] Courville T. Thompson B. (2001). Use of structure coefficients in published multiple regression articles: β is not enough. Educ. Psychol. Meas. 61, 229–2248. 10.1177/0013164401612006

[B12] Deming D. (2017). The growing importance of social skills in the labor market. Q. J. Econ. 132, 1593–1640. 10.1093/qje/qjx022

[B13] du Plessis G. de Bruin G. (2015). Using Rasch modelling to examine the international personality item pool (IPIP) values in action (VIA) measure of character strengths. J. Psychol. Afr. 25, 512–521. 10.1080/14330237.2015.1124603

[B14] Enders C. (2010). Applied Missing Data Analysis. Methodology in the Social Sciences. New York, NY: Guilford Press.

[B15] Estabrook R. Neale M. (2013). A comparison of factor score estimation methods in the presence of missing data: reliability and an application to nicotine dependence. Multivariate Behav. Res. 48, 1–27. 10.1080/00273171.2012.730072 24049215 PMC3773873

[B16] Fraley R. Vazire S. (2014). The N-pact factor: evaluating the quality of empirical journals with respect to sample size and statistical power. PLoS ONE 9:e109019. 10.1371/journal.pone.0109019 25296159 PMC4189949

[B17] Fuller W. (2006). Measurement Error Models. Hoboken, NJ: Wiley.

[B18] Gelman A. Hill J. Yajima M. (2012). Why we (usually) don't have to worry about multiple comparisons. J. Res. Educ. Eff. 5, 189–211. 10.1080/19345747.2011.618213

[B19] Harzer C. (2020). Fostering character strengths to promote thriving and flourishing in organizations. Organisationsberat Superv Coach 27, 37–50. 10.1007/s11613-020-00636-w

[B20] Hayes A. Coutts J. (2020). Use omega rather than cronbach's alpha for estimating reliability. But…. Commun. Methods Meas. 14, 1–24. 10.1080/19312458.2020.1718629

[B21] Hoogland J. (1999). The Robustness of Estimation Methods for Covariance Structure Analysis (Ph.D. thesis). University of Groningen, Groningen.

[B22] Hyslop R. Imbens G. (2001). Bias from classical and other forms of measurement error. J. Bus. Econ. Stat. 19, 475–481. 10.1198/07350010152596727

[B23] Junker B. Schofield L. (2012). The use of cognitive ability measures as explanatory variables in regression analysis. IZA J. Labor Econ. 1:4. 10.1186/2193-8997-1-4 26998417 PMC4798751

[B24] Kankaraš M. Suarez-Alvarez J. (2019). Assessment framework of the OECD Study on Social and Emotional Skills. Paris: OECD Publishing.

[B25] Laible M.-C. Anger S. Baumann M. (2020). Personality traits and further training. Front. Psychol. 11:510537. 10.3389/fpsyg.2020.510537 33304290 PMC7701053

[B26] Laukaityte I. Wiberg M. (2017). Using plausible values in secondary analysis in large-scale assessments. Commun. Stat. Theor. Methods 46, 11341-11357, 10.1080/03610926.2016.1267764

[B27] Laukaityte I. Wiberg M. (2018). Importance of sampling weights in multilevel modeling of international large-scale assessment data. Commun. Stat. Theory Methods 47, 4991–5012. 10.1080/03610926.2017.1383429

[B28] Lechner C. Anger S. Rammstedt B. (2019). Socio-emotional skills in education and beyond: recent evidence and future research avenues, in Research Handbook on the Sociology of Education, Research Handbooks in Sociology Series, ed Becker R. (Cheltenham: Edward Elgar), 427–453.

[B29] Lechner C. Bhaktha N. Groskurth K. Bluemke M. (2021). Why ability point estimates can be pointless: a primer on using skill measures from large-scale assessments in secondary analyses. Meas. Instrum. Soc. Sci. 3, 2. 10.1186/s42409-020-00020-5

[B30] Lechner C. Danner D. Rammstedt B. (2017). How is personality related to intelligence and achievement? A replication and extension of Borghans et al. and Salkever. Pers. Individ. Dif. 111, 86–91. 10.1016/j.paid.2017.01.040

[B31] Leite W. (2017). Practical Propensity Score Methods Using R. Thousand Oaks, CA: Sage Publications.

[B32] Little R. Rubin D. (2002). Statistical Analysis with Missing Data, 2nd Edn. Wiley Series in Probability and Statistics. Hoboken, NJ: Wiley.

[B33] Lu I. Thomas D. Zumbo B. (2005). Embedding IRT in structural equation models: a comparison with regression based on IRT scores. Struct. Equat. Model. Multidisciplinary J. 12, 263–277. 10.1207/s15328007sem1202_5

[B34] McDonald R. (1999). Test Theory: A Unified Treatment. Hillsdale, NJ: Lawrence Erlbaum Associates.

[B35] McNeish D. Wolf M. (2020). Thinking twice about sum scores. Behav. Res. Methods 52, 2287–2305. 10.3758/s13428-020-01398-0 32323277

[B36] Mislevy R. (1991). Randomization-based inference about latent variables from complex samples. Psychometrika 56, 177–196. 10.1007/BF02294457

[B37] Mislevy R. Beaton A. Kaplan B. Sheehan K. (1992). Estimating population characteristics from sparse matrix samples of item responses. J. Educ. Meas. 29, 133–161. 10.1111/j.1745-3984.1992.tb00371.x

[B38] Monseur C. Adams R. (2009). Plausible values: how to deal with their limitations. J. Appl. Meas. 10, 320–334. 19671992

[B39] Poon W.-Y. Wang H.-B. (2010). Analysis of a two-level structural equation model with missing data. Soc. Methods Res. 39, 25–55. 10.1177/0049124110371312

[B40] R Core Team (2020). R: A Language and Environment for Statistical Computing. Vienna: R Foundation for Statistical Computing.

[B41] Rabe-Hesketh S. Skrondal A. Pickles A. (2004). Generalized multilevel structural equation modeling. Psychometrika 69, 167–190. 10.1007/BF0229593928197954

[B42] Rammstedt B. Danner D. Lechner C. (2017). Personality, competencies, and life outcomes: results from the German PIAAC longitudinal study. Large-scale Assess. Educ. 5:2. 10.1186/s40536-017-0035-9

[B43] Rammstedt B. Lechner C. Danner D. (2021). Short forms do not fall short: a comparison of three (Extra-)short forms of the big five. Eur. J. Psychol. Assess. 37, 23–32. 10.1027/1015-5759/a000574

[B44] Rdz-Navarro K. (2019). Latent variables should remain as such: evidence from a Monte Carlo study. J. Gen Psychol. 146, 417–442. 10.1080/00221309.2019.1596064 31008695

[B45] Rhemthulla M. van Bork R. Borsboom D. (2020). Worse than measurement error: consequences of inappropriate latent variable measurement models. Psychol. Methods 25, 30–45. 10.1037/met0000220 31169371

[B46] Richards J. (1982). Standardized versus unstandardized regression weights. Appl. Psycho.l Meas. 6, 202–212. 10.1177/014662168200600206

[B47] Roberts B. Kuncel N. Shiner R. Caspi A. Goldberg L. (2007). The power of personality: the comparative validity of personality traits, socioeconomic status, and cognitive ability for predicting important life outcomes. Perspect. Psychol. Sci. 2, 313–345. 10.1111/j.1745-6916.2007.00047.x 26151971 PMC4499872

[B48] Robitzsch A. Grund S. (2021). miceadds: Some Additional Multiple Imputation Functions, Especially for 'mice'. Technical report. R package version 3. 11–16.

[B49] Robitzsch A. Kiefer T. Wu M. (2020). TAM: Test Analysis Modules. Technical report. R package version 3. 5–19.

[B50] Rosseel Y. (2012). lavaan: an R package for structural equation modeling. J. Stat. Softw. 48, 1–36. 10.18637/jss.v048.i0225601849

[B51] Schofield L. (2015). Correcting for measurement error in latent variables in used as predictors. Ann. Appl. Stat. 9, 2133–2152. 10.1214/15-AOAS877 26977218 PMC4787301

[B52] Sengewald M. Steiner P. Pohl S. (2018). When does measurement error in covariates impact causal effect estimates? analytic derivations of different scenarios and an empirical illustration. Br. J. Math. Stat. Psychol. 72, 244–270. 10.1111/bmsp.12146 30345554

[B53] Skrondal A. Laake P. (2001). Regression among factor scores. Psychometrika 66, 563–575. 10.1007/BF02296196

[B54] Soto C. John O. (2017). The next big five inventory (BFI-2): developing and assessing a hierarchical model with 15 facets to enhance bandwidth, fidelity, and predictive power. J. Pers. Soc. Psychol. 113, 117–143. 10.1037/pspp0000096 27055049

[B55] Soto C. J. Napolitano C. Sewell M. N. Yoon H. R. Roberts B. (2021). An integrative framework for conceptualizing and assessing social, emotional, and behavioral skills: the BESSI. PsyArXiv. 10.31234/osf.io/8m34z 35113631

[B56] Thalmayer A. Saucier G. Eigenhuis A. (2011). Comparative validity of brief to medium-length big five and big six personality questionnaires. Psychol Assess. 23, 995–1009. 10.1037/a0024165 21859221

[B57] von Davier M. (2010). Why sum scores may not tell us all about test takers. Newborn Infant Nurs. Rev. 10, 27–36. 10.1053/j.nainr.2009.12.011

[B58] von Davier M. Gonzalez E. Mislevy R. (2009). What are plausible values and why are they useful? IERI Monogr. Ser. 2, 9–36.

[B59] Wagner L. Holenstein M. Wepf H. Ruch W. (2020). Character strengths are related to students' achievement, flow experiences, and enjoyment in teacher-centered learning, individual, and group work beyond cognitive ability. Front. Psychol. 11:1324. 10.3389/fpsyg.2020.01324 32765332 PMC7378955

[B60] Warm T. (1989). Weighted likelihood estimation of ability in item response theory. Psychometrika 54, 427–450. 10.1007/BF02294627

[B61] Westfall J. Yarkoni T. (2016). Statistically controlling for confounding constructs is harder than you think. PLoS ONE 11:e0152719. 10.1371/journal.pone.0152719 27031707 PMC4816570

[B62] Wirth R. Edwards M. (2007). Item factor analysis: current approaches and future directions. Psychol. Methods 12, 58–79. 10.1037/1082-989X.12.1.58 17402812 PMC3162326

[B63] Wu M. (2005). The role of plausible values in large-scale surveys. Stud. Educ. Eval. 31, 114–128. 10.1016/j.stueduc.2005.05.005

