# Supplementary material for: To Score or Not to Score? A Simulation Study on the Performance of Test Scores, Plausible Values, and SEM, in Regression With Socio-Emotional Skill or Personality Scales as Predictors
Source: Front Psychol. 2021 Oct 15;12:679481. doi: 10.3389/fpsyg.2021.679481 (PMC8554300; doi:10.3389/fpsyg.2021.679481)
Supplement: Supplementary file 1 [file Data_Sheet_1.docx]

install.packages("pacman")

library(pacman)

p_load(tidyverse, dplyr, lavaan, TAM, purrr, broom, mice, miceadds, ggplot2,

reshape2, corrplot, update = FALSE

) # Set TRUE to update all

# Main functions used in the simulations study --------------------------------------

# Function to generate data when the factor loadings are strong

data_gen_high <- function(items, ss) {

if (items == 4) {

popmodel <- "V1 =~ 0.8 * X1 + 0.7 * X2 + 0.7 * X3 + 0.8 * X4

Y1 ~ 2.4 * V1 + 2 * V3

Y2 ~ 1.5 * V1 + 2.2 * V3

V1 ~~ 1*V1

X1 ~~ 0.36 * X1

X2 ~~ 0.51 * X2

X3 ~~ 0.51 * X3

X4 ~~ 0.36 * X4

V1 ~~ 0.3 * V3

Y1 ~~ 1*Y1

Y2 ~~ 1*Y2"

} else if (items == 8) {

popmodel <- "V1 =~ 0.8 * X1 + 0.7 * X2 + 0.7 * X3 + 0.8 * X4 +

0.8 * X5 + 0.7 * X6 + 0.7 * X7 + 0.8 * X8

Y1 ~ 2.4 * V1 + 2 * V3

Y2 ~ 1.5 * V1 + 2.2 * V3

V1 ~~ 1*V1

X1 ~~ 0.36 * X1

X2 ~~ 0.51 * X2

X3 ~~ 0.51 * X3

X4 ~~ 0.36 * X4

X5 ~~ 0.36 * X5

X6 ~~ 0.51 * X6

X7 ~~ 0.51 * X7

X8 ~~ 0.36 * X8

V1 ~~ 0.3 * V3

Y1 ~~ 1*Y1

Y2 ~~ 1*Y2"

} else {

popmodel <- "V1 =~ 0.8 * X1 + 0.7 * X2 + 0.7 * X3 + 0.8 * X4 +

0.8 * X5 + 0.7 * X6 + 0.7 * X7 + 0.8 * X8 +

0.8 * X9 + 0.7 * X10 + 0.7 * X11 + 0.8 * X12

Y1 ~ 2.4 * V1 + 2 * V3

Y2 ~ 1.5 * V1 + 2.2 * V3

V1 ~~ 1*V1

X1 ~~ 0.36 * X1

X2 ~~ 0.51 * X2

X3 ~~ 0.51 * X3

X4 ~~ 0.36 * X4

X5 ~~ 0.36 * X5

X6 ~~ 0.51 * X6

X7 ~~ 0.51 * X7

X8 ~~ 0.36 * X8

X9 ~~ 0.36 * X9

X10 ~~ 0.51 * X10

X11 ~~ 0.51 * X11

X12 ~~ 0.36 * X12

V1 ~~ 0.3 * V3

Y1 ~~ 1*Y1

Y2 ~~ 1*Y2"

}

# simulate data using above defined lavaan codes

dat <- simulateData(popmodel, sample.nobs = ss)

dat$ID <- 1:ss

# to convert continuous data to categorical

breaks <- c(-Inf, -1.645, -0.643, 0.643, 1.645, Inf)

cats <- function(x) {

cut(x, breaks = breaks, labels = c(1, 2, 3, 4, 5), ordered_result = FALSE)

}

num <- function(x) {

as.numeric(levels(x))[x]

}

dat <- dat %>% mutate_at(vars(matches("X")), cats)

dat <- dat %>% mutate_at(vars(matches("X")), num)

# extracting mean scores

dat <- dat %>% mutate(skill_ms =

rowMeans(select(dat, starts_with("X")), na.rm = TRUE))

# saving names of all items to be used in the CFA model

quest <- grep("X", names(dat), value = TRUE)

# generating lavaan code for CFA model

cfa_fs <- paste("skill =~ ", paste(quest, collapse = " + "))

# item factor analysis for EBM scores

cfa.fit1 <- sem(model = cfa_fs, data = dat, estimator = "WLSMV", std.lv = TRUE)

# extracting EBM scores

fscores <- lavPredict(object = cfa.fit1, type = "lv", method = "EBM")

idx <- lavInspect(cfa.fit1, "case.idx")

for (fs in colnames(fscores)) {

dat[idx, fs] <- fscores[, fs]

} # saving EBM scores in the dataframe

dat <- dat %>% rename(skill_ebm = skill)

cfa.fit2 <- sem(model = cfa_fs, data = dat, estimator = "MLR", std.lv = TRUE)

# extracting regression factor scores

fscores1 <- lavPredict(object = cfa.fit2, type = "lv", method = "regression")

idx <- lavInspect(cfa.fit2, "case.idx")

for (fs in colnames(fscores1)) {

dat[idx, fs] <- fscores1[, fs]

} # saving regression factor scores in the dataframe

dat <- dat %>% rename(skill_rs = skill)

}

# Function to generate data when the factor loadings are mixed

data_gen_mixed <- function(items, ss) {

if (items == 4) {

popmodel <- "V1 =~ 0.5 * X1 + 0.6 * X2 + 0.7 * X3 + 0.8 * X4

Y1 ~ 2.4 * V1 + 2 * V3

Y2 ~ 1.5 * V1 + 2.2 * V3

V1 ~~ 1*V1

X1 ~~ 0.75 * X1

X2 ~~ 0.64 * X2

X3 ~~ 0.51 * X3

X4 ~~ 0.36 * X4

V1 ~~ 0.3 * V3

Y1 ~~ 1*Y1

Y2 ~~ 1*Y2"

} else if (items == 8) {

popmodel <- "V1 =~ 0.4 * X1 + 0.5 * X2 + 0.6 * X3 + 0.6 * X4 +

0.7 * X5 + 0.7 * X6 + 0.8 * X7 + 0.9 * X8

Y1 ~ 2.4 * V1 + 2 * V3

Y2 ~ 1.5 * V1 + 2.2 * V3

V1 ~~ 1*V1

X1 ~~ 0.84 * X1

X2 ~~ 0.75 * X2

X3 ~~ 0.64 * X3

X4 ~~ 0.64 * X4

X5 ~~ 0.51 * X5

X6 ~~ 0.51 * X6

X7 ~~ 0.36 * X7

X8 ~~ 0.19 * X8

V1 ~~ 0.3 * V3

Y1 ~~ 1*Y1

Y2 ~~ 1*Y2"

} else {

popmodel <- "V1 =~ 0.4 * X1 + 0.5 * X2 + 0.5 * X3 + 0.6 * X4 +

0.6 * X5 + 0.6 * X6 + 0.7 * X7 + 0.7 * X8 +

0.7 * X9 + 0.8 * X10 + 0.8 * X11 + 0.9 * X12

Y1 ~ 2.4 * V1 + 2 * V3

Y2 ~ 1.5 * V1 + 2.2 * V3

V1 ~~ 1*V1

X1 ~~ 0.84 * X1

X2 ~~ 0.75 * X2

X3 ~~ 0.75 * X3

X4 ~~ 0.64 * X4

X5 ~~ 0.64 * X5

X6 ~~ 0.64 * X6

X7 ~~ 0.51 * X7

X8 ~~ 0.51 * X8

X9 ~~ 0.51 * X9

X10 ~~ 0.36 * X10

X11 ~~ 0.36 * X11

X12 ~~ 0.19 * X12

V1 ~~ 0.3 * V3

Y1 ~~ 1*Y1

Y2 ~~ 1*Y2"

}

dat <- simulateData(popmodel, sample.nobs = ss)

dat$ID <- 1:ss

breaks <- c(-Inf, -1.645, -0.643, 0.643, 1.645, Inf)

cats <- function(x) {

cut(x, breaks = breaks, labels = c(1, 2, 3, 4, 5), ordered_result = FALSE)

}

num <- function(x) {

as.numeric(levels(x))[x]

}

dat <- dat %>% mutate_at(vars(matches("X")), cats)

dat <- dat %>% mutate_at(vars(matches("X")), num)

dat <- dat %>% mutate(skill_ms =

rowMeans(select(dat, starts_with("X")), na.rm = TRUE))

quest <- grep("X", names(dat), value = TRUE)

cfa_fs <- paste("skill =~ ", paste(quest, collapse = " + "))

cfa.fit1 <- sem(model = cfa_fs, data = dat, estimator = "WLSMV", std.lv = TRUE)

fscores <- lavPredict(object = cfa.fit1, type = "lv", method = "EBM")

idx <- lavInspect(cfa.fit1, "case.idx")

for (fs in colnames(fscores)) {

dat[idx, fs] <- fscores[, fs]

}

dat <- dat %>% rename(skill_ebm = skill)

cfa.fit2 <- sem(model = cfa_fs, data = dat, estimator = "MLR", std.lv = TRUE)

fscores1 <- lavPredict(object = cfa.fit2, type = "lv", method = "regression")

idx <- lavInspect(cfa.fit2, "case.idx")

for (fs in colnames(fscores1)) {

dat[idx, fs] <- fscores1[, fs]

}

dat <- dat %>% rename(skill_rs = skill)

}

# Function to generate data when the factor loadings are weak

data_gen_weak <- function(items, ss) {

if (items == 4) {

popmodel <- "V1 =~ 0.4 * X1 + 0.5 * X2 + 0.4 * X3 + 0.5 * X4

Y1 ~ 2.4 * V1 + 2 * V3

Y2 ~ 1.5 * V1 + 2.2 * V3

V1 ~~ 1*V1

X1 ~~ 0.84 * X1

X2 ~~ 0.75 * X2

X3 ~~ 0.84 * X3

X4 ~~ 0.75 * X4

V1 ~~ 0.3 * V3

Y1 ~~ 1*Y1

Y2 ~~ 1*Y2"

} else if (items == 8) {

popmodel <- "V1 =~ 0.4 * X1 + 0.5 * X2 + 0.4 * X3 + 0.5 * X4 +

0.4 * X5 + 0.5 * X6 + 0.4 * X7 + 0.5 * X8

Y1 ~ 2.4 * V1 + 2 * V3

Y2 ~ 1.5 * V1 + 2.2 * V3

V1 ~~ 1*V1

X1 ~~ 0.84 * X1

X2 ~~ 0.75 * X2

X3 ~~ 0.84 * X3

X4 ~~ 0.75 * X4

X5 ~~ 0.84 * X5

X6 ~~ 0.75 * X6

X7 ~~ 0.84 * X7

X8 ~~ 0.75 * X8

V1 ~~ 0.3 * V3

Y1 ~~ 1*Y1

Y2 ~~ 1*Y2"

} else {

popmodel <- "V1 =~ 0.4 * X1 + 0.5 * X2 + 0.4 * X3 + 0.5 * X4 +

0.4 * X5 + 0.5 * X6 + 0.4 * X7 + 0.5 * X8 +

0.4 * X9 + 0.5 * X10 + 0.4 * X11 + 0.5 * X12

Y1 ~ 2.4 * V1 + 2 * V3

Y2 ~ 1.5 * V1 + 2.2 * V3

V1 ~~ 1*V1

X1 ~~ 0.84 * X1

X2 ~~ 0.75 * X2

X3 ~~ 0.84 * X3

X4 ~~ 0.75 * X4

X5 ~~ 0.84 * X5

X6 ~~ 0.75 * X6

X7 ~~ 0.84 * X7

X8 ~~ 0.75 * X8

X9 ~~ 0.84 * X9

X10 ~~ 0.75 * X10

X11 ~~ 0.84 * X11

X12 ~~ 0.75 * X12

V1 ~~ 0.3 * V3

Y1 ~~ 1*Y1

Y2 ~~ 1*Y2"

}

dat <- simulateData(popmodel, sample.nobs = ss)

dat$ID <- 1:ss

breaks <- c(-Inf, -1.645, -0.643, 0.643, 1.645, Inf)

cats <- function(x) {

cut(x, breaks = breaks, labels = c(1, 2, 3, 4, 5), ordered_result = FALSE)

}

num <- function(x) {

as.numeric(levels(x))[x]

}

dat <- dat %>% mutate_at(vars(matches("X")), cats)

dat <- dat %>% mutate_at(vars(matches("X")), num)

dat <- dat %>% mutate(skill_ms =

rowMeans(select(dat, starts_with("X")), na.rm = TRUE))

quest <- grep("X", names(dat), value = TRUE)

cfa_fs <- paste("skill =~ ", paste(quest, collapse = " + "))

cfa.fit1 <- sem(model = cfa_fs, data = dat, estimator = "WLSMV", std.lv = TRUE)

fscores <- lavPredict(object = cfa.fit1, type = "lv", method = "EBM")

idx <- lavInspect(cfa.fit1, "case.idx")

for (fs in colnames(fscores)) {

dat[idx, fs] <- fscores[, fs]

}

dat <- dat %>% rename(skill_ebm = skill)

cfa.fit2 <- sem(model = cfa_fs, data = dat, estimator = "MLR", std.lv = TRUE)

fscores1 <- lavPredict(object = cfa.fit2, type = "lv", method = "regression")

idx <- lavInspect(cfa.fit2, "case.idx")

for (fs in colnames(fscores1)) {

dat[idx, fs] <- fscores1[, fs]

}

dat <- dat %>% rename(skill_rs = skill)

}

# Function to run GPCM IRT model

irt_mod <- function(items, dt) {

mod.gpcm <- tam.mml.2pl(resp = dt[, c(1:items)], irtmodel = "GPCM")

}

# Function to extract WLE and EAP scores from the GPCM IRT model fit above

add_wle_eap <- function(dt, irt.mod) {

dt <- dt %>% mutate(

skill_wle = tam.wle(irt.mod)$theta,

skill_eap = IRT.factor.scores(irt.mod)$EAP

)

}

# Function to run regression with standardized mean scores and

# to extract the estimated parameters

ms_reg <- function(dat) {

mod1 <- lm(Y1 ~ scale(skill_ms) + V3, data = dat)

mod2 <- lm(Y2 ~ scale(skill_ms) + V3, data = dat)

param1 <- tidy(mod1)

param2 <- tidy(mod2)

param <- list(y1 = param1, y2 = param2)

}

# Function to run regression with EBM scores and to extract the estimated parameters

ebm_reg <- function(dat) {

mod1 <- lm(Y1 ~ scale(skill_ebm) + V3, data = dat)

mod2 <- lm(Y2 ~ scale(skill_ebm) + V3, data = dat)

param1 <- tidy(mod1)

param2 <- tidy(mod2)

param <- list(y1 = param1, y2 = param2)

}

# Function to run regression with regression factor scores and

# to extract the estimated parameters

rs_reg <- function(dat) {

mod1 <- lm(Y1 ~ scale(skill_rs) + V3, data = dat)

mod2 <- lm(Y2 ~ scale(skill_rs) + V3, data = dat)

param1 <- tidy(mod1)

param2 <- tidy(mod2)

param <- list(y1 = param1, y2 = param2)

}

# Function to run regression with WLE scores and to extract the estimated parameters

wle_reg <- function(dat) {

mod1 <- lm(Y1 ~ scale(skill_wle) + V3, data = dat)

mod2 <- lm(Y2 ~ scale(skill_wle) + V3, data = dat)

param1 <- tidy(mod1)

param2 <- tidy(mod2)

param <- list(y1 = param1, y2 = param2)

}

# Function to run regression with EAP scores and to extract the estimated parameters

eap_reg <- function(dat) {

mod1 <- lm(Y1 ~ scale(skill_eap) + V3, data = dat)

mod2 <- lm(Y2 ~ scale(skill_eap) + V3, data = dat)

param1 <- tidy(mod1)

param2 <- tidy(mod2)

param <- list(y1 = param1, y2 = param2)

}

# Function to run SEM model and to extract the estimated parameters

sem_reg <- function(dat) {

quest <- grep("X", names(dat), value = TRUE)

sem_for <- paste(

"skill =~ ", paste(quest, collapse = " + "), "\n", "Y1 ~ skill + V3",

"\n", "Y2 ~ skill + V3", "\n", "skill ~~ V3"

)

fit1 <- sem(model = sem_for, data = dat, estimator = "MLR", std.lv = TRUE)

param <- tidy(fit1)

}

# Function to save 10 plausible values and run &

# save regression estimates using multiple imputation framework

pvc_reg <- function(irt.mod, items, dat) {

xsi0 <- irt.mod$xsi$xsi

xsi.fixed <- cbind(seq(1, length(xsi0)), xsi0)

cv <- dat[, c("Y1", "Y2", "V3")]

mod2b <- tam.mml(resp = dat[, c(1:items)], B = irt.mod$B, xsi.fixed = xsi.fixed,

Y = cv)

pvc <- tam.pv(mod2b, nplausible = 10, ntheta = 500, normal.approx = TRUE)

# using all other variables in the population model

Y <- dat[, c("ID", "Y1", "Y2", "V3")]

pvnames <- c("pvc")

datlist1 <- tampv2datalist(pvc, pvnames = pvnames, Y = Y, Y.pid = "ID")

mids1 <- miceadds::datalist2mids(datlist1)

mod1 <- with(mids1, lm(Y1 ~ pvc + V3))

mod2 <- with(mids1, lm(Y2 ~ pvc + V3))

param1 <- as.data.frame(summary(pool(mod1)))

param2 <- as.data.frame(summary(pool(mod2)))

param <- list(y1 = param1, y2 = param2)

}

# Function to compute correlations among test scores and the outcome variables

cor_scores <- function(dat) {

correl <- cor(dat[, c(

"skill_ms", "skill_ebm", "skill_rs", "skill_wle",

"skill_eap", "Y1", "Y2"

)])

}

# Function to extract regression coefficient of the skill for outcome variable y1

coef_y1 <- function(reg) {

cy1 <- reg[["y1"]][["estimate"]][2]

}

# Function to extract regression coefficient of the skill for outcome variable y2

coef_y2 <- function(reg) {

cy2 <- reg[["y2"]][["estimate"]][2]

}

# Function to extract path coefficient of the skill to outcome variable y1

coef_sem_y1 <- function(csem.reg) {

cy1 <- csem.reg$estimate[csem.reg$term == "Y1 ~ skill"]

}

# Function to extract path coefficient of the skill to outcome variable y2

coef_sem_y2 <- function(csem.reg) {

cy2 <- csem.reg$estimate[csem.reg$term == "Y2 ~ skill"]

}

# Function to extract regression coefficient of the covariate for outcome variable y1

cov_coefy1 <- function(reg) {

cy1 <- reg[["y1"]][["estimate"]][3]

}

# Function to extract regression coefficient of the covariate for outcome variable y2

cov_coefy2 <- function(reg) {

cy2 <- reg[["y2"]][["estimate"]][3]

}

# Function to extract path coefficient of the covariate to outcome variable y1

cov_coefsem_y1 <- function(csem.reg) {

cy1 <- csem.reg$estimate[csem.reg$term == "Y1 ~ V3"]

}

# Function to extract path coefficient of the covariate to outcome variable y2

cov_coefsem_y2 <- function(csem.reg) {

cy2 <- csem.reg$estimate[csem.reg$term == "Y2 ~ V3"]

}

# Simulation Study Tibble -----------------------------------------------------------

# Main tibble with condition specifications

items <- c(4, 8, 12)

ss <- c(300, 1000)

reps <- 1:500

sim <- as_tibble(crossing(items, ss, reps))

# Simulation tibble for items with high factor loadings -----------------------------

set.seed(123456)

sim_high <- sim %>%

mutate(

dt = pmap(list(items, ss), data_gen_high),

irt.mod = pmap(list(items, dt), irt_mod),

dat = pmap(list(dt, irt.mod), add_wle_eap)

) %>%

select(-c(dt))

saveRDS(sim_high, "sim_high_with_data.rds")

sim_high <- sim_high %>% mutate(

sms.reg = pmap(list(dat), ms_reg),

ebm.reg = pmap(list(dat), ebm_reg),

rs.reg = pmap(list(dat), rs_reg),

wle.reg = pmap(list(dat), wle_reg),

eap.reg = pmap(list(dat), eap_reg),

sem.reg = pmap(list(dat), sem_reg),

pvc.reg = pmap(list(irt.mod, items, dat), pvc_reg),

cor.scores = pmap(list(dat), cor_scores)

)

sim_high <- sim_high %>% select(-c(irt.mod, dat))

# saving tibble in RDS format after deleting heavier objects

saveRDS(sim_high, "sim_high_reg_results.rds")

# Simulation tibble with items having mixed factor loadings -------------------------

set.seed(123456)

sim_mixed <- sim %>%

mutate(

dt = pmap(list(items, ss), data_gen_mixed),

irt.mod = pmap(list(items, dt), irt_mod),

dat = pmap(list(dt, irt.mod), add_wle_eap)

) %>%

select(-c(dt))

saveRDS(sim_mixed, "sim_mixed_with_data.rds")

sim_mixed <- sim_mixed %>% mutate(

sms.reg = pmap(list(dat), ms_reg),

ebm.reg = pmap(list(dat), ebm_reg),

rs.reg = pmap(list(dat), rs_reg),

wle.reg = pmap(list(dat), wle_reg),

eap.reg = pmap(list(dat), eap_reg),

sem.reg = pmap(list(dat), sem_reg),

pvc.reg = pmap(list(irt.mod, items, dat), pvc_reg),

cor.scores = pmap(list(dat), cor_scores)

)

sim_mixed <- sim_mixed %>% select(-c(irt.mod, dat))

saveRDS(sim_mixed, "sim_mixed_reg_results.rds")

# Simulation tibble for items with weak factor loadings -----------------------------

set.seed(123456)

sim_weak <- sim %>%

mutate(

dt = pmap(list(items, ss), data_gen_weak),

irt.mod = pmap(list(items, dt), irt_mod),

dat = pmap(list(dt, irt.mod), add_wle_eap)

) %>%

select(-c(dt))

saveRDS(sim_weak, "sim_weak_with_data.rds")

sim_weak <- sim_weak %>% mutate(

sms.reg = pmap(list(dat), ms_reg),

ebm.reg = pmap(list(dat), ebm_reg),

rs.reg = pmap(list(dat), rs_reg),

wle.reg = pmap(list(dat), wle_reg),

eap.reg = pmap(list(dat), eap_reg),

sem.reg = pmap(list(dat), sem_reg),

pvc.reg = pmap(list(irt.mod, items, dat), pvc_reg),

cor.scores = pmap(list(dat), cor_scores)

)

sim_weak <- sim_weak %>% select(-c(irt.mod, dat))

saveRDS(sim_weak, "sim_weak_reg_results.rds")

# Analysis of simulation study results ----------------------------------------------

# Merging files ---------------------------------------------------------------------

sim_high <- readRDS("sim_high_reg_results.rds")

sim_high <- sim_high %>% mutate(factor_loadings = "high")

sim_mixed <- readRDS("sim_mixed_reg_results.rds")

sim_mixed <- sim_mixed %>% mutate(factor_loadings = "mixed")

sim_weak <- readRDS("sim_weak_reg_results.rds")

sim_weak <- sim_weak %>% mutate(factor_loadings = "weak")

sim_tib <- rbind.data.frame(sim_high, sim_mixed, sim_weak)

saveRDS(sim_tib, "sim_tib_full_reg.rds") # save the merged data file

# Bias in regression coefficients ---------------------------------------------------

# Bias in skill ---------------------------------------------------------------------

simtib <- readRDS("sim_tib_full_reg.rds")

# Etracting all regression and path coefficients of the skill

simtib <- simtib %>% mutate(

sms.y1 = unlist(pmap(list(reg = sms.reg), coef_y1)),

ebm.y1 = unlist(pmap(list(reg = ebm.reg), coef_y1)),

rs.y1 = unlist(pmap(list(reg = rs.reg), coef_y1)),

wle.y1 = unlist(pmap(list(reg = wle.reg), coef_y1)),

eap.y1 = unlist(pmap(list(reg = eap.reg), coef_y1)),

pvc.y1 = unlist(pmap(list(reg = pvc.reg), coef_y1)),

sms.y2 = unlist(pmap(list(reg = sms.reg), coef_y2)),

ebm.y2 = unlist(pmap(list(reg = ebm.reg), coef_y2)),

rs.y2 = unlist(pmap(list(reg = rs.reg), coef_y2)),

wle.y2 = unlist(pmap(list(reg = wle.reg), coef_y2)),

eap.y2 = unlist(pmap(list(reg = eap.reg), coef_y2)),

pvc.y2 = unlist(pmap(list(reg = pvc.reg), coef_y2)),

sem.y1 = unlist(pmap(list(csem.reg), coef_sem_y1)),

sem.y2 = unlist(pmap(list(csem.reg), coef_sem_y2))

)

# reduced tibble only for outcome Y1

simtiby1 <- simtib %>% select(ss, factor_loadings, items, reps, ends_with("y1"))

# convert data form wide to long format

simtiby1 <- melt(simtiby1, measure.vars = 5:11, variable.name = "method")

simtiby1 <- simtiby1 %>%

rename(coeff = value, Items = items, Sample_Size = ss) %>%

mutate(method = recode(method,

sms.y1 = "SMS", ebm.y1 = "EBM",

rs.y1 = "RFS", wle.y1 = "WLE",

eap.y1 = "EAP", pvc.y1 = "PV",

sem.y1 = "SEM"

)) # Rename to appear accordingly in graphs and tables

simtiby1 <- simtiby1 %>% mutate(

bias = coeff - 2.4,

pcb = (bias / 2.4) * 100

) # calculate bias based on population model

simtiby1 <- simtiby1 %>% mutate(reg_coeff = "Higher")

# reduced tibble only for outcome Y2

simtiby2 <- simtib %>% select(ss, factor_loadings, items, reps, ends_with("y2"))

simtiby2 <- melt(simtiby2, measure.vars = 5:11, variable.name = "method")

# convert data form wide to long format

simtiby2 <- simtiby2 %>%

rename(coeff = value, Items = items, Sample_Size = ss) %>%

mutate(method = recode(method,

sms.y2 = "SMS", ebm.y2 = "EBM",

rs.y2 = "RFS", wle.y2 = "WLE",

eap.y2 = "EAP", pvc.y2 = "PV", sem.y2 = "SEM"

))

simtiby2 <- simtiby2 %>% mutate(

bias = coeff - 1.5,

pcb = (bias / 1.5) * 100

) # calculate bias based on population model

simtiby2 <- simtiby2 %>% mutate(reg_coeff = "Lower")

# merging the two reduced tibbles

simtib_skill <- rbind.data.frame(simtiby1, simtiby2)

simtib_skill$Sample_Size <- as.factor(simtib_skill$Sample_Size)

simtib_skill$Items <- as.factor(simtib_skill$Items)

simtib_skill$factor_loadings <- as.factor(simtib_skill$factor_loadings)

simtib_skill$reg_coeff <- as.factor(simtib_skill$reg_coeff)

# Table of mean regression coefficient of the skill of 500 simulations

# under different conditions for Y1

coef_tab1 <- simtiby1 %>%

group_by(Sample_Size, factor_loadings, Items, method) %>%

summarize_at("coeff", list(mean = mean)) %>%

ungroup() %>%

spread(., key = method, value = mean)

# Table of mean percentage bias in regression coefficient of the skill of

# 500 simulations under different conditions for Y1

bias_taby1 <- simtiby1 %>%

group_by(Sample_Size, factor_loadings, Items, method) %>%

summarize_at("pcb", list(mean = mean)) %>%

ungroup() %>%

spread(., key = method, value = mean)

# Table of mean regression coefficient of the skill of 500 simulations

# under different conditions for Y2

coef_tab2 <- simtiby2 %>%

group_by(Sample_Size, factor_loadings, Items, method) %>%

summarize_at("coeff", list(mean = mean)) %>%

ungroup() %>%

spread(., key = method, value = mean)

# Table of mean percentage bias in regression coefficient of the skill of

# 500 simulations under different conditions for Y2

bias_taby2 <- simtiby2 %>%

group_by(Sample_Size, factor_loadings, Items, method) %>%

summarize_at("pcb", list(mean = mean)) %>%

ungroup() %>%

spread(., key = method, value = mean)

# Specification for graphs ----------------------------------------------------------

item.lab <- c("4 Items", "8 Items", "12 Items")

names(item.lab) <- c(4, 8, 12)

ss.lab <- c("n = 300", "n = 1000")

names(ss.lab) <- c(300, 1000)

# Defining plot theme

plot_theme <- function() {

theme(

axis.text = element_text(face = "bold", color = "black"),

axis.title = element_text(face = "bold", color = "black"),

strip.text = element_text(face = "bold", color = "black"),

strip.background = element_rect(colour = "black", fill = "white"),

legend.title = element_text(face = "bold", color = "black"),

legend.text = element_text(face = "bold", color = "black"),

legend.key = element_rect(fill = "white", colour = "black"),

plot.title = element_text(size = 11, face = "bold", hjust = 0.5),

panel.grid.major = element_blank(),

panel.grid.minor = element_blank(),

panel.background = element_blank(),

panel.border = element_rect(colour = "black", fill = NA)

)

}

# Plot of % bias when eelative size of the relationship of the skill with the outcome

# is higher than the covariate

ggplot(data = simtiby1, aes(x = method, y = pcb, fill = factor_loadings)) +

geom_boxplot(errorbar.draw = TRUE, outlier.color = NA) +

scale_y_continuous(limit = c(-75, 50), breaks = c(-75, -50, -25, 0, 25, 50)) +

scale_fill_manual(values = c("#CC6677", "#DDCC77", "#88CCEE"),

labels = c("High", "Mixed", "Low")) +

facet_grid(Sample_Size ~ Items,

labeller = labeller(Items = item.lab, Sample_Size = ss.lab)) +

geom_hline(yintercept = 0, lty = "dashed") +

xlab("Method") +

ylab("Percentage Bias") +

labs(fill = "Factor Loadings") +

ggtitle("A: Relative size of the relationship of the skill with the outcome is

higher than the covariate") +

plot_theme()

# Plot of % bias when eelative size of the relationship of the skill with the outcome

# is lower than the covariate

ggplot(data = simtiby2, aes(x = method, y = pcb, fill = factor_loadings)) +

geom_boxplot(errorbar.draw = TRUE, outlier.color = NA) +

scale_y_continuous(limit = c(-75, 50), breaks = c(-75, -50, -25, 0, 25, 50)) +

scale_fill_manual(values = c("#CC6677", "#DDCC77", "#88CCEE"),

labels = c("High", "Mixed", "Low")) +

facet_grid(Sample_Size ~ Items,

labeller = labeller(Items = item.lab, Sample_Size = ss.lab)) +

geom_hline(yintercept = 0, lty = "dashed") +

xlab("Method") +

ylab("Percentage Bias") +

labs(fill = "Factor Loadings") +

ggtitle("B: Relative size of the relationship of the skill with the outcome is

lower than the covariate") +

plot_theme()

# Bias in covariate -----------------------------------------------------------------

simtib_cov <- readRDS("sim_tib_full_reg.rds")

# Etracting all regression and path coefficients of the covariate

simtib_cov <- simtib_cov %>% mutate(

sms.cov.y1 = unlist(pmap(list(reg = sms.reg), cov_coefy1)),

ebm.cov.y1 = unlist(pmap(list(reg = ebm.reg), cov_coefy1)),

rs.cov.y1 = unlist(pmap(list(reg = rs.reg), cov_coefy1)),

wle.cov.y1 = unlist(pmap(list(reg = wle.reg), cov_coefy1)),

eap.cov.y1 = unlist(pmap(list(reg = eap.reg), cov_coefy1)),

pvc.cov.y1 = unlist(pmap(list(reg = pvc.reg), cov_coefy1)),

sms.cov.y2 = unlist(pmap(list(reg = sms.reg), cov_coefy2)),

ebm.cov.y2 = unlist(pmap(list(reg = ebm.reg), cov_coefy2)),

rs.cov.y2 = unlist(pmap(list(reg = rs.reg), cov_coefy2)),

wle.cov.y2 = unlist(pmap(list(reg = wle.reg), cov_coefy2)),

eap.cov.y2 = unlist(pmap(list(reg = eap.reg), cov_coefy2)),

pvc.cov.y2 = unlist(pmap(list(reg = pvc.reg), cov_coefy2)),

sem.cov.y1 = unlist(pmap(list(csem.reg), cov_coefsem_y1)),

sem.cov.y2 = unlist(pmap(list(csem.reg), cov_coefsem_y2))

)

simtib_covy1 <- simtib_cov %>% select(ss, factor_loadings, items, reps,

ends_with("y1"))

simtib_covy1 <- melt(simtib_covy1, measure.vars = 5:11, variable.name = "method")

simtib_covy1 <- simtib_covy1 %>%

rename(cov_coeff = value, Items = items, Sample_Size = ss) %>%

mutate(method = recode(method,

sms.cov.y1 = "SMS", ebm.cov.y1 = "EBM",

rs.cov.y1 = "RFS", wle.cov.y1 = "WLE",

eap.cov.y1 = "EAP", pvc.cov.y1 = "PV",

sem.cov.y1 = "SEM"

))

simtib_covy1 <- simtib_covy1 %>% mutate(

bias = cov_coeff - 2,

pcb = (bias / 2) * 100

)

simtib_covy1 <- simtib_covy1 %>% mutate(reg_cov_coeff = "Higher")

simtib_covy2 <- simtib_cov %>% select(ss, factor_loadings, items, reps,

ends_with("y2"))

simtib_covy2 <- melt(simtib_covy2, measure.vars = 5:11, variable.name = "method")

simtib_covy2 <- simtib_covy2 %>%

rename(cov_coeff = value, Items = items, Sample_Size = ss) %>%

mutate(method = recode(method,

sms.cov.y2 = "SMS", ebm.cov.y2 = "EBM",

rs.cov.y2 = "RFS", wle.cov.y2 = "WLE",

eap.cov.y2 = "EAP", pvc.cov.y2 = "PV", sem.cov.y2 = "SEM"

))

simtib_covy2 <- simtib_covy2 %>% mutate(

bias = cov_coeff - 2.2,

pcb = (bias / 2.2) * 100

)

simtib_covy2 <- simtib_covy2 %>% mutate(reg_cov_coeff = "Lower")

simtib_cov_skill <- rbind.data.frame(simtib_covy1, simtib_covy2)

simtib_cov_skill$Sample_Size <- as.factor(simtib_cov_skill$Sample_Size)

simtib_cov_skill$Items <- as.factor(simtib_cov_skill$Items)

simtib_cov_skill$factor_loadings <- as.factor(simtib_cov_skill$factor_loadings)

simtib_cov_skill$reg_cov_coeff <- as.factor(simtib_cov_skill$reg_cov_coeff)

cov_coef_tab1 <- simtib_covy1 %>%

group_by(Sample_Size, factor_loadings, Items, method) %>%

summarize_at("cov_coeff", list(mean = mean)) %>%

ungroup() %>%

spread(., key = method, value = mean)

bias_taby1 <- simtib_covy1 %>%

group_by(Sample_Size, factor_loadings, Items, method) %>%

summarize_at("pcb", list(mean = mean)) %>%

ungroup() %>%

spread(., key = method, value = mean)

cov_coef_tab2 <- simtib_covy2 %>%

group_by(Sample_Size, factor_loadings, Items, method) %>%

summarize_at("cov_coeff", list(mean = mean)) %>%

ungroup() %>%

spread(., key = method, value = mean)

bias_taby2 <- simtib_covy2 %>%

group_by(Sample_Size, factor_loadings, Items, method) %>%

summarize_at("pcb", list(mean = mean)) %>%

ungroup() %>%

spread(., key = method, value = mean)

item.lab <- c("4 Items", "8 Items", "12 Items")

names(item.lab) <- c(4, 8, 12)

ss.lab <- c("n = 300", "n = 1000")

names(ss.lab) <- c(300, 1000)

plot_theme <- function() {

theme(

axis.text = element_text(face = "bold", color = "black"),

axis.title = element_text(face = "bold", color = "black"),

strip.text = element_text(face = "bold", color = "black"),

strip.background = element_rect(colour = "black", fill = "white"),

legend.title = element_text(face = "bold", color = "black"),

legend.text = element_text(face = "bold", color = "black"),

legend.key = element_rect(fill = "white", colour = "black"),

plot.title = element_text(size = 11, face = "bold", hjust = 0.5),

panel.grid.major = element_blank(),

panel.grid.minor = element_blank(),

panel.background = element_blank(),

panel.border = element_rect(colour = "black", fill = NA)

)

}

ggplot(data = simtib_covy1, aes(x = method, y = pcb, fill = factor_loadings)) +

geom_boxplot(errorbar.draw = TRUE, outlier.color = NA) +

scale_y_continuous(limit = c(-40, 40), breaks = c(-40, -20, 0, 20, 40)) +

scale_fill_manual(values = c("#CC6677", "#DDCC77", "#88CCEE"),

labels = c("High", "Mixed", "Low")) +

facet_grid(Sample_Size ~ Items,

labeller = labeller(Items = item.lab, Sample_Size = ss.lab)) +

geom_hline(yintercept = 0, lty = "dashed") +

xlab("Method") +

ylab("Percentage Bias") +

labs(fill = "Factor Loadings") +

ggtitle("A: Relative size of the relationship of the skill with the outcome is

higher than the covariate") +

plot_theme()

ggplot(data = simtib_covy2, aes(x = method, y = pcb, fill = factor_loadings)) +

geom_boxplot(errorbar.draw = TRUE, outlier.color = NA) +

scale_y_continuous(limit = c(-40, 40), breaks = c(-40, -20, 0, 20, 40)) +

scale_fill_manual(values = c("#CC6677", "#DDCC77", "#88CCEE"),

labels = c("High", "Mixed", "Low")) +

facet_grid(Sample_Size ~ Items,

labeller = labeller(Items = item.lab, Sample_Size = ss.lab)) +

geom_hline(yintercept = 0, lty = "dashed") +

xlab("Method") +

ylab("Percentage Bias") +

labs(fill = "Factor Loadings") +

ggtitle("B: Relative size of the relationship of the skill with the outcome is

lower than the covariate") +

plot_theme()

# Correlation between scores --------------------------------------------------------

cormat <- readRDS("sim_tib_full_reg.rds")

cormat <- cormat %>% select(-c(ends_with(".reg"), ms.reg.y))

cordat <- apply(simplify2array(cormat$cor.scores), 1:2, mean)

rownames(cordat) <- c("SMS", "EBM", "RFS", "WLE", "EAP", "Y1", "Y2")

colnames(cordat) <- c("SMS", "EBM", "RFS", "WLE", "EAP", "Y1", "Y2")

corrplot.mixed(cordat,

lower.col = "black", tl.col = "black", cl.lim = c(0.5, 1),

outline = TRUE,

upper.col = colorRampPalette(c("#CCFF66", "#CCCC66", "#FFFF00", "#DDCC77",

"#CC6677"))(100)

)
